# Reactive polymer confinement enables low-temperature Bi_2_O_2_CO_3_/ZnO heterojunction assembly for visible-light photocatalysis

**DOI:** 10.1039/d6ra08383h

**Published:** 2026-11-09

**Authors:** Xiangfei Chen, Yinji Lu, Yi Jin, Jie Li, Wentao Zhu, Yanwen Lv, Jiangang Yu

**Affiliations:** a Department of Chemical and Material Engineering, Quzhou College of Technology No. 18 Jiangyuan Road Quzhou 324002 Zhejiang China; b College of Chemical and Material Engineering, Quzhou University Quzhou 324000 China qzlyw@qzc.edu.cn jgyu@qzc.edu.cn

## Abstract

Achieving controlled inorganic phase dispersion and intimate heterointerface assembly within polymer-inorganic hybrid systems under mild processing conditions remains challenging, as polymer matrices are commonly adopted as passive supports and only provide limited regulation over inorganic phase organization. Herein, polyvinylmethyldimethoxysilane (PVMDMS) is employed as a reactive confinement medium to direct the low-temperature assembly of Bi_2_O_2_CO_3_/ZnO heterojunctions. During sol–gel processing and subsequent aging at 80 °C, the hydrolysis and condensation of PVMDMS generate a cross-linked polymer matrix, hereafter denoted as PV, concurrently with inorganic phase formation, which restricts particle diffusion and aggregation and promotes intimate nanoscale contact between Bi_2_O_2_CO_3_ nanosheets and granular ZnO domains. This polymer-regulated assembly process produces a hierarchical nanosheet-granule architecture with accessible mesoporosity and enhanced interfacial coupling. Structural, spectroscopic, and photoelectrochemical analyses demonstrate that the regulated heterointerface contributes to improved visible-light utilization, suppressed charge-carrier recombination, and accelerated interfacial charge transfer. Consequently, the optimized Bi_2_O_2_CO_3_/ZnO composite incorporated within PV, designated as 5Bi-ZnO@PV with a nominal *n*_Bi_/(*n*_Zn_ + *n*_Bi_) precursor molar ratio of 5%, achieves 92.6% methyl orange removal within 40 min under 450 nm irradiation, with an apparent rate constant of 0.0519 min^−1^, approximately 7.3 times that of the Bi-free ZnO/PV reference composite (ZnO@PV). Scavenger and electron paramagnetic resonance (EPR) analyses confirm that ˙O_2_^−^ and h^+^ are the dominant reactive species, and 79.9% removal efficiency is retained after five cycles of operation. These results establish a polymer-confinement strategy for coupling macromolecular network evolution with inorganic heterointerface assembly, and demonstrate how polymer-regulated hierarchical organization can be translated into enhanced photoactive function.

## Abstract 2

Polymer-confined Bi_2_O_2_CO_3_/ZnO heterojunctions assembled at 80 °C promote interfacial charge transfer. The composite removes 92.6% of methyl orange in 40 min under 450 nm light and retains 79.9% removal after five cycles.

## Graphical abstract

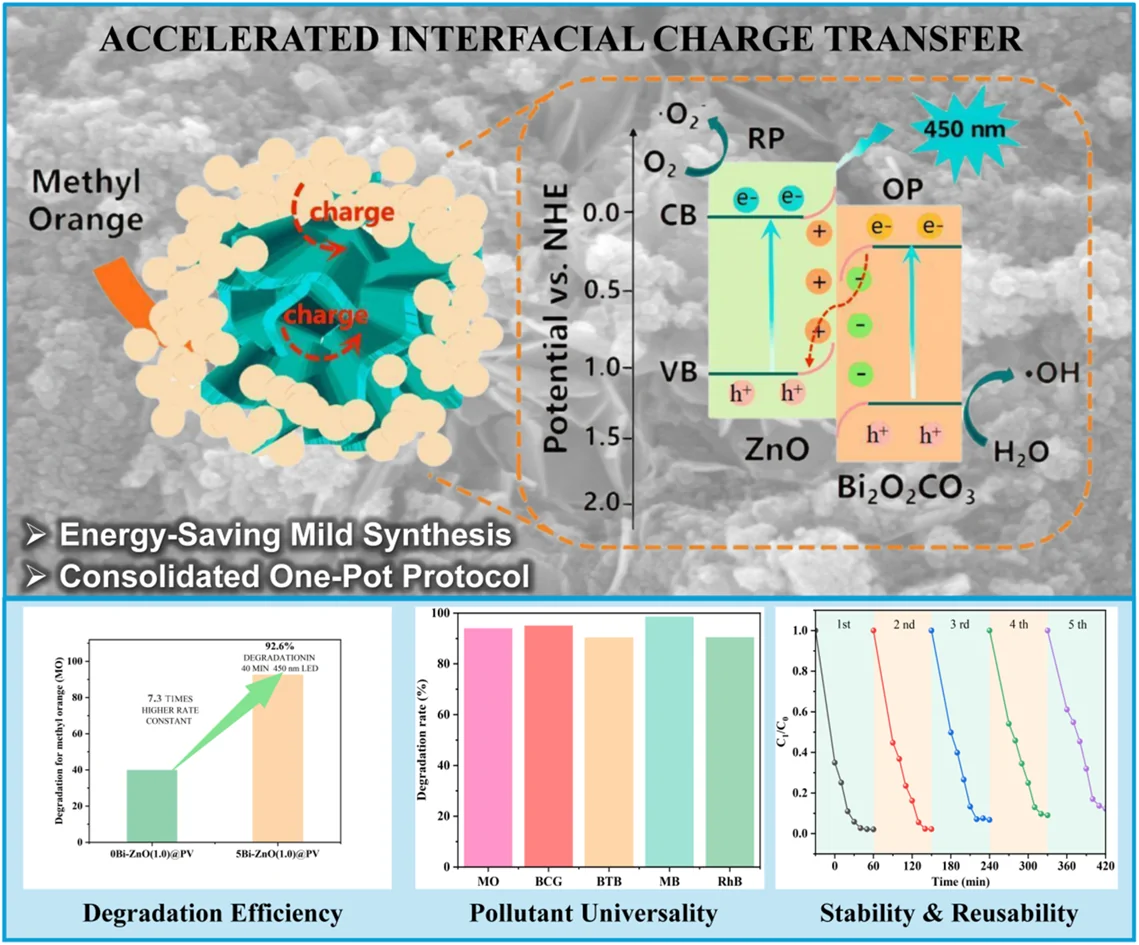

## 1. Introduction

Driven by rapid industrialization, the escalating discharge of organic pollutants into aquatic environments poses a severe threat to global ecosystems and human health.^1,2^ The urgency of this crisis is underscored by recent World Health Organization statistics, which reveal that over two billion people rely on contaminated water sources, with waterborne infections accounting for approximately 1.5 million deaths annually.^3^ Among these contaminants, a diverse array of organic pollutants, particularly synthetic dyes and pharmaceutical residues, demonstrate pronounced resistance to conventional biological and physical degradation processes.^4^ Consequently, traditional wastewater remediation techniques, such as coagulation,^5^ biological processes,^6^ and chemical oxidation,^7^ are increasingly inadequate.^8^ These methodologies are frequently hindered by substantial limitations, including significant operational expenditures and considerable energy consumption, as well as the generation of large volumes of residual sludge that necessitates subsequent energy-intensive treatment.^9,10^

In recent years, semiconductor-based photocatalysis has emerged as a promising, environmentally benign, and sustainable technology for ecological remediation.^11,12^ By harnessing abundant and renewable solar energy, this process can effectively mineralize toxic organic contaminants into harmless inorganic molecules. Among various photocatalytic materials, zinc oxide (ZnO) attracts considerable research interest owing to its cost-effectiveness, inherent non-toxicity, biocompatibility, and high electron mobility.^13,14^ Nevertheless, the practical deployment of pristine ZnO is constrained by two intrinsic limitations: a wide bandgap that restricts its optical absorption primarily to the ultraviolet region, and the rapid recombination of photogenerated electron–hole pairs that severely impedes charge transfer kinetics.^15–17^ Consequently, engineering ZnO to extend its light absorption into the visible spectrum and suppress charge carrier recombination remains a task of paramount importance. To overcome these intrinsic bottlenecks, the construction of semiconductor heterojunctions has emerged as a highly effective strategy.^18^ Coupling ZnO with a suitable secondary semiconductor establishes a built-in electric field at the interface, thereby providing a strong driving force for the spatial separation of photogenerated electron–hole pairs. In this regard, bismuth subcarbonate (Bi_2_O_2_CO_3_), a distinctive layered material composed of alternating [Bi_2_O_2_]^2+^ and CO_3_^2−^ layers, has garnered considerable attention.^19^ Its unique internal static electric field and inherent visible-light responsiveness make it an ideal candidate for heterojunction coupling. To maximize its catalytic potential, various surface decoration and heterojunction strategies have been extensively explored. For instance, a recent investigation elucidated the interfacial charge transfer dynamics of a g-C_3_N_4_ surface-decorated Bi_2_O_2_CO_3_ photocatalyst synthesized *via* a hydrothermal method.^20^ Supported by first-principles simulations, the optimized 10 wt% g-C_3_N_4_/Bi_2_O_2_CO_3_ composite exhibited exceptional simulated solar-light-driven photocatalytic degradation performance toward a range of broad-spectrum antibiotics (*e.g.*, tetracycline, tetracycline hydrochloride, and oxytetracycline) and organic dyes, even within complex real aqueous matrices. This enhanced performance is primarily attributable to accelerated charge transfer rates and inhibited electron–hole recombination. Furthermore, to elucidate the specific synergy between ZnO and Bi_2_O_2_CO_3_, recent pioneering research has detailed the sonochemical synthesis of Bi_2_O_2_CO_3_/ZnO heterojunctions, which demonstrate superior redox capacities.^21^ Comprehensive optoelectronic and morphological analyses revealed that the optimized composite achieved an outstanding 97.2% degradation efficiency for Rhodamine B within 180 min under natural sunlight irradiation. This significant improvement is ascribed to the intimate interfacial deposition of Bi_2_O_2_CO_3_ onto ZnO, broadened solar absorption, and the successful construction of a direct S-scheme charge transportation pathway.

Despite these substantial advancements in Bi_2_O_2_CO_3_-based systems, prevailing fabrication strategies, most notably the aforementioned hydrothermal and sonochemical procedures, frequently suffer from intricate multi-step protocols, high energy consumption, and insufficient long-term structural stability, which collectively hinder their scalable industrial application.^22,23^ Moreover, a critical and predominantly unresolved challenge remains: the seamless integration of active inorganic dual-phase heterojunctions into a flexible organic polymer matrix. Such integration is desirable as it effectively prevents nanoparticle agglomeration and enriches surface active sites, particularly if achieved through a facile, single-step, and low-temperature process.^24^ Polyvinylmethyldimethoxysilane (PVMDMS) extends beyond its conventional role as a passive physical support, functioning as a nanoreactor that critically facilitates the formation of heterojunctions.^25^ The cross-linked siloxane network inherent to PVMDMS exerts a pronounced spatial confinement effect, which effectively restricts excessive crystal growth and suppresses the undesirable aggregation of inorganic phases.^26,27^ Furthermore, the inherent functional groups within the polymer backbone facilitate precise surface energy modulation, acting as a molecular template to guide the seamless interfacial assembly between Bi_2_O_2_CO_3_ nanoplates and ZnO nanoparticles. In this work, Bi_2_O_2_CO_3_/ZnO composites are prepared in a polyvinylmethyldimethoxysilane (PVMDMS)-derived matrix through sol–gel assisted precipitation followed by aging at 80 °C. PVMDMS serves as the polymer precursor, and the matrix formed through hydrolysis and condensation of its methoxysilane groups is denoted PV. This preparation route is investigated for assembling Bi_2_O_2_CO_3_ and ZnO within a polymer-derived network. By varying the Bi precursor content, we examine changes in the crystalline phase composition, nanosheet-granule morphology, and pore structure of the resulting composites. Structural and textural characterization is combined with optical, photoelectrochemical, and photocatalytic measurements to investigate the associations among composition, microstructure, charge-carrier behavior, and methyl orange removal under 450 nm irradiation. The study therefore focuses on polymer-assisted low-temperature assembly and the observed structure–performance relationships in Bi_2_O_2_CO_3_/ZnO@PV composites.

## 2. Experimental section

### 2.1 Materials

All chemical reagents were used as received without further purification. Deionized (DI) water was used throughout the experiments for the preparation of all aqueous solutions. Nafion perfluorosulfonic acid polymer dispersion D520 (5%) was purchased from Suzhou Sinero Technology Co., Ltd sodium hydroxide (NaOH, 96.0%), ethanol (C_2_H_5_OH, 99%), and acetic acid (CH_3_COOH, 99.5%) were procured from Xilong Chemical Co., Ltd methanol (CH_3_OH, 99.9%), 2-chlorophenol (2-CP, 99%), levofloxacin (LVX, 98%), potassium persulfate (K_2_S_2_O_8_, 99.9%), and anhydrous sodium sulfate (Na_2_SO_4_, 99%) were obtained from Shanghai Maclin Biochemical Technology Co., Ltd Zinc acetate (Zn(CH_3_COO)_2_, 99.5%), bismuth nitrate pentahydrate (Bi(NO_3_)_3_·5H_2_O, 99.99%), methyl orange (MO, 99%), methylene blue (MB, 98%), bisphenol A (BPA, 99%), vinylmethyldimethoxysilane (VMDMS, 98%), and di-*tert*-butyl peroxide (DTBP, 98%) were purchased from Shanghai Titan Scientific Co., Ltd Rhodamine B (RhB, 99%), bromocresol green (BCG, 99%), and bromothymol blue (BTB, 99%) were purchased from Sinopharm Chemical Reagent Co., Ltd isopropanol (IPA, 99.7%), ammonium oxalate (AO, 99%), and *p*-benzoquinone (*p*-BQ, 99%) were sourced from Shanghai Aladdin Biochemical Technology Co., Ltd.

### 2.2 Preparation of PVMDMS

PVMDMS was synthesized *via* a bulk polymerization approach. Briefly, the initiator DTBP (1.00 g, 6.8 mmol) was dissolved in the monomer VMDMS (9.00 g, 68.1 mmol). The mixture was magnetically stirred at 300 rpm for 10 min at 25 °C within a 50 mL polytetrafluoroethylene (PTFE)-lined stainless–steel autoclave. Subsequently, the sealed autoclave was placed in a convection oven that had been preheated and equilibrated at 120 °C and was kept in the oven for 48 h. Upon natural cooling to 25 °C, the resulting viscous polymeric product was collected, transferred into a 50 mL amber glass vial, and stored at −20 °C prior to subsequent use.^28^

### 2.3 Catalyst preparation

#### 2.3.1 Synthesis of the pristine polymer matrix

The pristine PVMDMS-derived cross-linked polymer matrix (PV) was prepared *via* a hydrolytic condensation process. Initially, PVMDMS was dissolved in a co-solvent mixture of methanol and ethanol. Acetic acid was gradually added to the PVMDMS solution until an apparent pH of 6 was reached. The resulting mixture was allowed to stand undisturbed for 2 h. Subsequently, an aqueous tetramethylammonium hydroxide (TMAOH) solution (25%) was added dropwise until a final apparent pH of 8 was reached. The precursor mixture was then thermally aged at 80 °C for 3 h. Upon completion, the reaction vessel was allowed to cool naturally to 25 °C. The resultant solid product was collected and purified by washing consecutively with ethanol and DI water (three times each) to remove unreacted reagents. Finally, the purified PV sample was dried at 70 °C for 12 h.

#### 2.3.2 Preparation of ZnO(*m*)@PV composites

A series of Bi-free ZnO(*m*)@PV composites was prepared by varying the zinc acetate feed mass, where *m* denotes the mass of zinc acetate in grams. The values of *m* were 0.2, 0.4, 0.6, 0.8, 1.0, and 1.2. Zinc acetate was dissolved in 30 mL of methanol and mixed with a solution containing 0.4 g of PVMDMS in 2.0 g of ethanol. After stirring for 5 min, 1.22 g of 1 mol L^−1^ aqueous acetic acid was added dropwise, followed by stirring at 25 °C for 60 min. An aqueous NaOH solution containing 27.2 mmol of NaOH in 8 g of deionized water was then added dropwise, followed by stirring for 15 min. No bismuth precursor was added. The resulting suspension was aged at 80 °C for 3 h. After cooling, the precipitate was washed three times each with ethanol and deionized water and dried at 70 °C for 12 h.

#### 2.3.3 Synthesis of Bi_2_O_2_CO_3_/ZnO@PV heterojunctions

The *x*Bi-ZnO@PV composites were prepared through sol–gel processing followed by low-temperature aging at 80 °C, where *x* denotes the nominal 100*n*_Bi_/(*n*_Zn_ + *n*_Bi_) precursor molar percentage. In a typical procedure, 1.0 g of zinc acetate was dissolved in 30 mL of methanol to form solution A. Concurrently, 0.4 g of PVMDMS was dissolved in 2.0 g of ethanol to form solution B. Solutions A and B were thoroughly mixed and stirred for 5 minutes to ensure homogeneous dispersion. Subsequently, 1.22 g of a 1 mol L^−1^ aqueous acetic acid solution was added dropwise to the mixture. The solution was stirred at 25 °C for 60 min to facilitate the complete hydrolysis of the methoxy groups in the PVMDMS polymer. Following the hydrolysis phase, a specific amount of bismuth nitrate pentahydrate (*e.g.*, 139.1 mg, 0.287 mmol for the 3Bi-ZnO@PV sample), pre-dissolved in dilute nitric acid, was slowly dripped into the solution. Next, an alkaline solution containing 27.2 mmol of NaOH dissolved in 8.0 g of DI water was added dropwise under continuous stirring for 15 min. After the reagent addition and stirring steps, the resulting precursor suspension was transferred to a reaction vessel and aged in an oven at 80 °C for 3 h. After the reaction, the vessel was allowed to cool naturally to 25 °C. The obtained solid precipitates were collected and washed three times each with absolute ethanol and DI water to remove residual ions. Finally, the product was dried in an oven at 70 °C for 12 h. By systematically varying the initial mass of the bismuth precursor (the percentage of the Bi/(Zn + Bi) molar ratio), a series of nanocomposites denoted as ZnO@PV, 1Bi-ZnO@PV, 3Bi-ZnO@PV, 5Bi-ZnO@PV, and 7Bi-ZnO@PV were successfully prepared.

### 2.4 Characterization

Fourier transform infrared (FT-IR) spectra were recorded on a Nicolet 6700 spectrometer using the KBr pellet technique (resolution of 2 cm^−1^, 32 scans, 500–4000 cm^−1^). The specific surface area and porous structure of the catalysts were evaluated *via* N_2_ adsorption–desorption isotherms at −196 °C using a Micromeritics ASAP 2460 analyzer. X-ray photoelectron spectroscopy (XPS) measurements were conducted on a Thermo Fisher Scientific K-Alpha system to analyze the surface chemical composition, with all binding energies calibrated to the adventitious C 1s peak at 284.6 eV. The crystal structures were determined by X-ray diffraction (XRD, Bruker D8 Advance) equipped with Cu Kα radiation (40 kV, 40 mA) at a scanning rate of 5°·min^−1^.

Morphological and microstructural features were examined using a scanning electron microscope (SEM, Zeiss Sigma 360) and a transmission electron microscope/high-resolution transmission electron microscopy (TEM/HRTEM, FEI Tecnai G2 F30 S-Twin). Elemental distribution mappings of the nanocomposites were acquired *via* energy-dispersive X-ray spectroscopy (EDS). UV-vis diffuse reflectance spectra (DRS) were collected on a PerkinElmer Lambda 650 S spectrophotometer equipped with an integrating sphere, using BaSO_4_ as the reflectance standard to evaluate optical absorption properties. Electron paramagnetic resonance (EPR) signals were recorded on a Bruker A300 spectrometer to investigate the electronic properties and detect reactive oxygen species, utilizing methanol and aqueous dispersions for trapping superoxide radicals (˙O_2_^−^) and hydroxyl radicals (˙OH), respectively. Steady-state photoluminescence (PL) emission spectra were recorded using a spectrofluorometer (Hitachi High-Tech Science Corporation, F-4700) at a fixed excitation wavelength of 370 nm. VB-XPS spectra were acquired using a Thermo Scientific K-Alpha spectrometer with Al Kα radiation (1486.68 eV), a pass energy of 50 eV, and an energy step of 0.1 eV. Binding energies were charge-corrected by referencing the C 1s peak to 284.8 eV. UPS measurements were performed using a Thermo Scientific Nexsa spectrometer with a He I source (21.22 eV), a pass energy of 2.0 eV, and an energy step of 0.020 eV. A sample bias of −5 V was applied during acquisition of the secondary-electron cutoff spectrum.

### 2.5 Photocatalytic performance

Photocatalytic tests were performed using a blue LED with a nominal emission maximum of 450 nm and a rated power of 50 W. This wavelength was selected to evaluate light-driven pollutant removal in the blue region of the visible spectrum and to compare the prepared samples under the same illumination conditions. The additional visible-region absorption observed in the diffuse reflectance spectra motivated evaluation in this wavelength range; however, the wavelength selection does not imply that 450 nm is optimal or that both semiconductor components undergo direct band-to-band excitation. In a standard procedure, 150 mg of the photocatalyst was dispersed into 50 mL of a MO aqueous solution (13 mg L^−1^). Prior to illumination, the suspension was continuously stirred at 500 rpm for 30 min in the dark to establish an adsorption–desorption equilibrium between the photocatalyst surface and the MO molecules. Upon initiating the light irradiation, 4 mL aliquots were extracted at 10 min intervals. The sampled aliquots were promptly centrifuged and filtered through a 0.22 µm membrane to remove the catalyst particles. The residual concentration of the MO solution was then determined by measuring the absorbance at its maximum absorption wavelength (463 nm) using a UV-vis spectrophotometer. The photocatalytic degradation efficiency was calculated using the following equation:Degradation rate (%) = (*A*_0_ − *A*_*t*_)/*A*_0_ × 100%where *A*_0_ represents the initial absorbance of the MO solution at adsorption equilibrium, and *A*_*t*_ represents the absorbance after *t* min of photocatalytic degradation.

### 2.6 Photoelectrochemical measurements

The working electrodes were fabricated by dispersing 10 mg of the catalyst powder and 20 µL of a perfluorinated resin solution (5 wt% Nafion, Suzhou Sinero Technology) in 200 µL of anhydrous ethanol. This mixture was ultrasonicated for 90 min to formulate a homogeneous catalyst ink. Subsequently, 45 µL of the resulting suspension was drop-cast onto the conductive surface of an indium tin oxide (ITO) glass substrate, carefully covering a defined active area of 1 × 1 cm^2^. The coated electrodes were then allowed to dry naturally at room temperature. All photoelectrochemical (PEC) evaluations were performed in a standard three-electrode configuration using a 0.5 M aqueous sodium sulfate solution as the electrolyte. The prepared catalyst-coated ITO glass served as the working electrode, alongside a platinum plate counter electrode and an Ag/AgCl reference electrode. Electrochemical impedance spectroscopy (EIS) measurements were recorded over a frequency range of 0.1 Hz to 100 kHz with an AC amplitude perturbation of 5 mV. A 300 W xenon lamp was utilized to irradiate the working electrode. Transient photocurrent responses (*I*–*t* curves) were recorded under chopped illumination to evaluate the charge transfer dynamics under alternating light and dark cycles.

## 3. Results and discussion

### 3.1 Synthesis and structural design

The *x*Bi-ZnO@PV heterojunction composites were prepared through sol–gel processing followed by low-temperature aging at 80 °C (Fig. 1). Utilizing the *in situ* hydrolysis of PVMDMS as a dynamic nanoreactor^25^ avoids the high-temperature calcination typically needed to crystallize metal oxides. The hydrolyzed siloxane network provides abundant Si–OH groups, which interact with metal precursors through coordination and electrostatic interactions. During condensation, these interactions spatially immobilize Zn^2+^ and Bi^3+^ species, restricting long-range diffusion and crystal growth, thereby inducing nanoscale confinement. The formation of CO_3_^2−^ may be attributed to the use of high-concentration NaOH and the presence of bismuth salts, coupled with the system's extreme sensitivity to airborne CO_2_, which led to the *in situ* formation of carbonate-containing components during thermal aging.^29^ This low-temperature (80 °C) aging process preserves the polymer shell's functional groups and spatially confines the nanoparticles, preventing agglomeration and ensuring intimate interfacial contact.

**Fig. 1:**
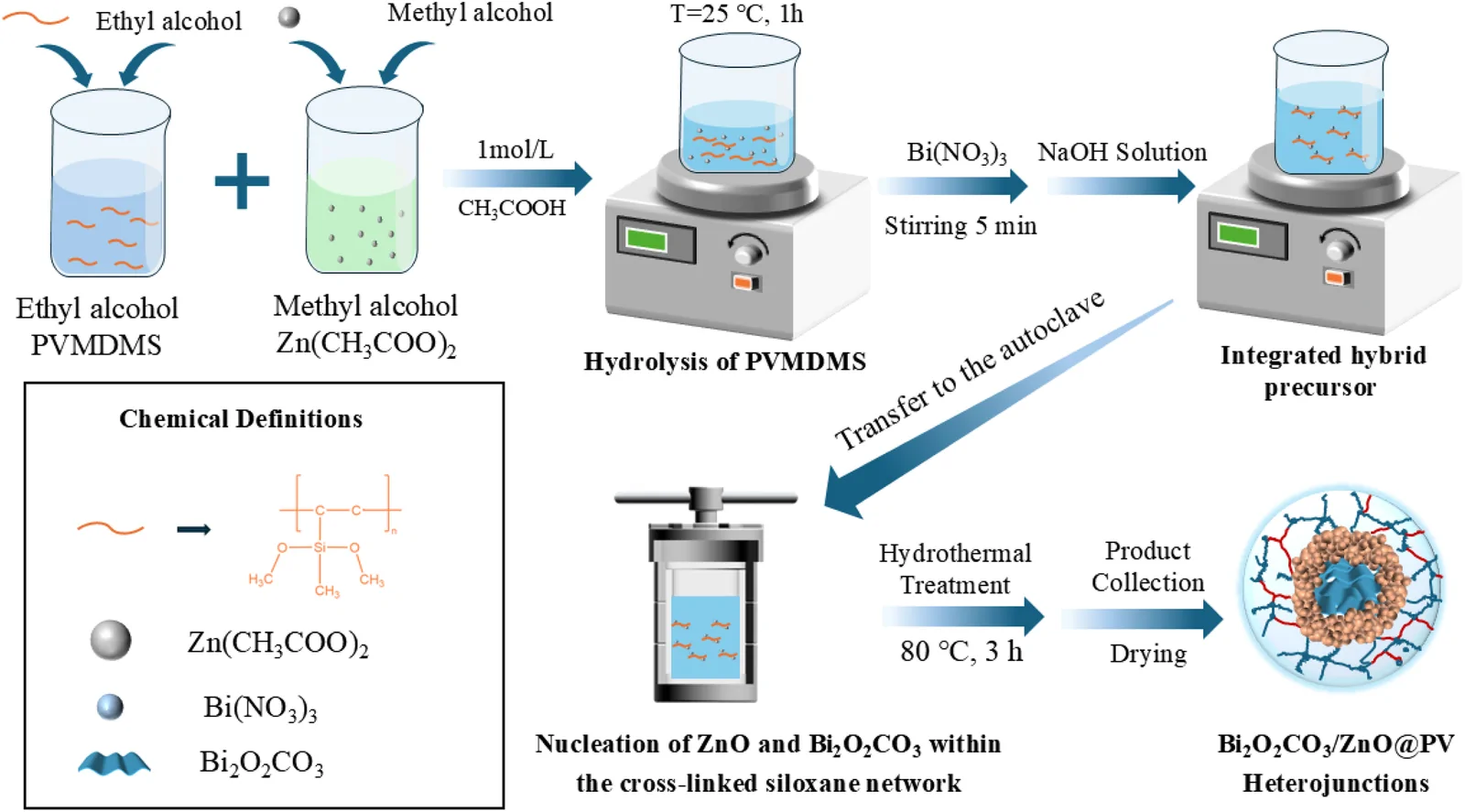
Schematic illustration of the reactive polymer-confinement-enabled assembly of Bi_2_O_2_CO_3_/ZnO@PV heterojunctions through sol–gel processing followed by low-temperature aging at 80 °C.

### 3.2 Structural, chemical, and thermal characterization

The successful formation of the heterojunctions was comprehensively verified through spectroscopic, crystallographic, and thermal analyses. The FTIR spectra (Fig. 2a) exhibit Si–O–Si stretching bands^4^ at approximately 1000–1100 cm^−1^ and C–H stretching bands in the 2750–3000 cm^−1^ region,^30^ consistent with the polymer-derived matrix.^31,32^ However, distinct Bi–O and Zn–O bands cannot be reliably resolved in the present spectra. The enlarged 800–900 cm^−1^ region (Fig. 2b) contains potentially overlapping matrix and carbonate-related contributions and is therefore not used as independent evidence for Bi–O/Zn–O bond formation. Raman spectroscopy provides complementary information on the vibrational characteristics of the composites (Fig. 2c). The broad band around 2900 cm^−1^ is associated with C–H stretching vibrations of the polymer matrix. The feature at 437 cm^−1^ is consistent with the *E*_2_ (high) mode of wurtzite ZnO. Following Bi incorporation, the bands at 162 cm^−1^ and 1067 cm^−1^ are assigned to Bi–O related lattice vibrations and the symmetric stretching vibration of CO_3_^2−^, respectively, based on comparison with reported Bi_2_O_2_CO_3_ spectra.^33,34^ The additional low-frequency features are not regarded as specific evidence of oxygen vacancies because their origins cannot be uniquely established from the present Raman data.

**Fig. 2:**
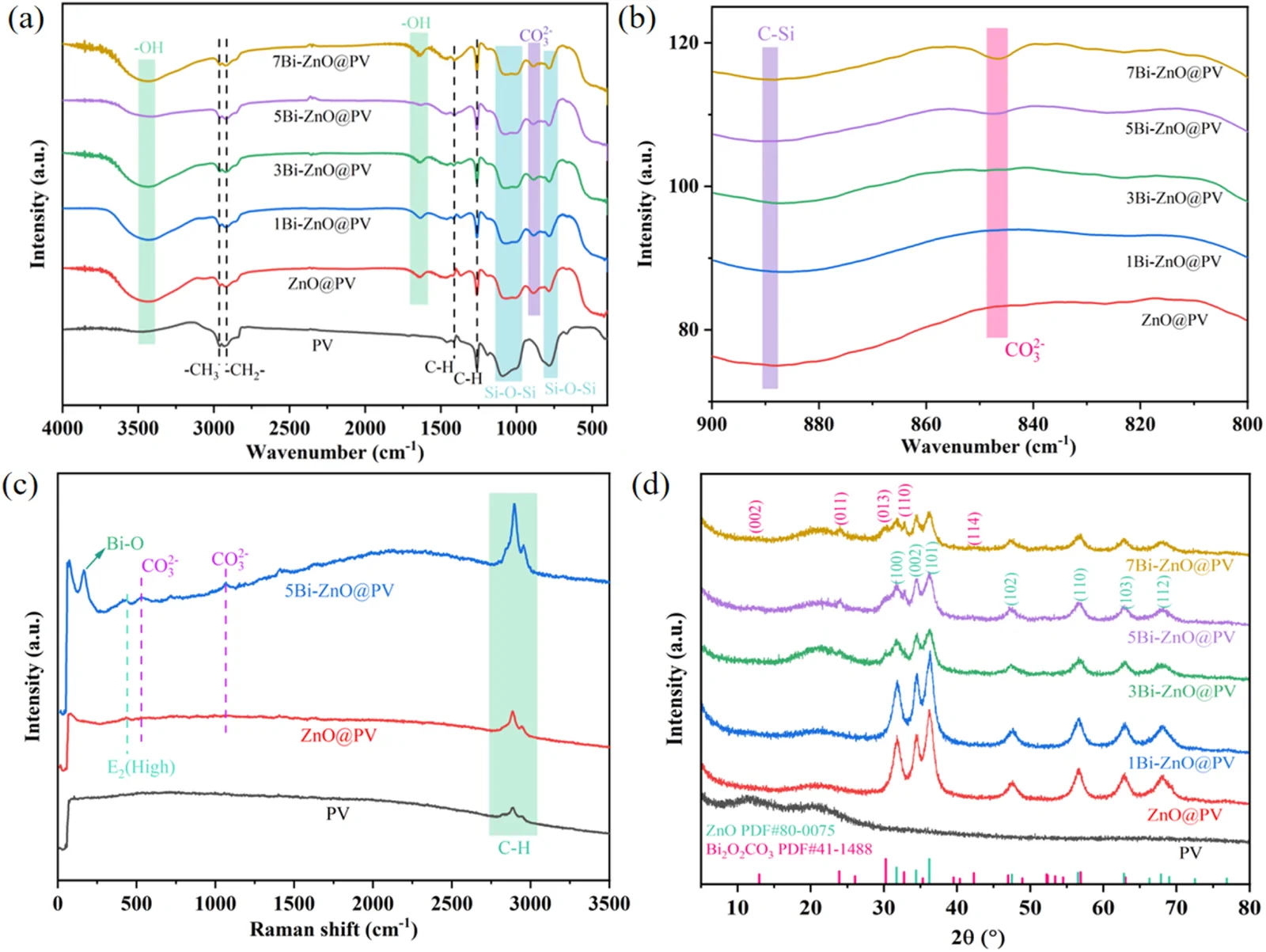
(a) FT-IR spectra of the PV, ZnO@PV, 1Bi-ZnO@PV, 3Bi-ZnO@PV, 5Bi-ZnO@PV, and 7Bi-ZnO@PV samples; (b) enlarged FT-IR spectra of the corresponding samples in the range of 800–900 cm^−1^; (c) Raman spectra of PV, ZnO@PV, and 5Bi-ZnO@PV, with the measured positions of the assigned vibrational bands indicated; (d) XRD patterns of the PV, ZnO@PV, 1Bi-ZnO@PV, 3Bi-ZnO@PV, 5Bi-ZnO@PV, and 7Bi-ZnO@PV samples.

The XRD patterns are presented in Fig. 2d. The PV reference exhibits a broad amorphous background, whereas ZnO@PV shows reflections consistent with wurtzite ZnO. In the Bi-containing composites, reflections at 2*θ* = 31.7° and 34.4° are assigned to the (013) and (110) of Bi_2_O_2_CO_3_ using PDF No. #41-1488. Differences in relative intensities may arise from preferred orientation of the nanosheets, phase proportions, and overlapping reflections. These intensity differences are distinguished from peak-position discrepancies, which were evaluated separately during phase identification.^35^

XPS was employed to characterize the surface chemical composition of the composite materials. The XPS survey spectra (Fig. 3a–f) corroborate the presence of all anticipated elements. For both the ZnO@PV and 5Bi-ZnO@PV samples (Fig. 3a), distinct peaks corresponding to Zn 2p, O 1s, C 1s, as well as Si 2p and Si 2s originating from the PV substrate are clearly discernible. In contrast, the survey XPS spectrum of the 5Bi-ZnO@PV sample reveals additional characteristic peaks attributed to Bi 4d and Bi 4f (Fig. 3b), thereby providing direct evidence for the successful incorporation of the Bi_2_O_2_CO_3_ phase into the ZnO@PV composite system. High-resolution spectra provide further insight into the specific chemical microenvironments. The Si 2p spectrum (Fig. 3c) showed a peak at approximately 102.06 eV, which is consistent with the polysiloxane network. The C 1s spectra (Fig. 3e) could be deconvoluted into three peaks located at ∼284.8, ∼286.8, and ∼288.8 eV, corresponding to C–C/C–H, C–O, and O=C–O bonds, respectively. The peak at ∼288.8 eV is a typical indicator of carbonate ions (CO_3_^2−^), further verifying the presence of the Bi_2_O_2_CO_3_ modification. The high-resolution Bi 4f spectrum (Fig. 3b) contains a dominant doublet at 159.26 and 164.56 eV, assigned to Bi^3+^ 4f_7/2_ and 4f_5/2_, respectively. Additional lower-binding-energy components are fitted at 157.54 and 162.73 eV. Their positions are close to reported metallic Bi signals and are therefore tentatively assigned to Bi^0^.^36^ The lower-binding-energy O 1s contribution near 530.5–530.7 eV is consistent with metal–oxygen environments associated with Zn–O and Bi–O. The higher-binding-energy contributions in the approximately 531.5–532.6 eV region may contain overlapping signals from carbonate, hydroxyl, siloxane, and other oxygen-containing surface species.^37^ These contributions cannot be uniquely assigned to oxygen vacancies, and the O 1s spectra are therefore not used to determine an oxygen-vacancy concentration.^38^ Upon the introduction of Bi_2_O_2_CO_3_, discernible alterations in both the binding energies and relative proportions of the surface oxygen species were observed. More importantly, compared to the pristine ZnO@PV matrix, the high-resolution Zn 2p spectra of the 5Bi-ZnO@PV composite (Fig. 3d) revealed a pronounced positive shift. The upshift of the Zn 2p_3/2_ and Zn 2p_1/2_ peaks, from 1021.68 eV and 1044.69 eV to 1021.98 eV and 1045.00 eV, respectively, signifies a reduction in the electron cloud density surrounding the Zn atoms. Collectively, these spectral evolutions offer spectroscopic proof for the strong electronic interaction and directional interfacial charge transfer across the ZnO/Bi_2_O_2_CO_3_ heterojunction,^39^ thereby laying a solid structural foundation for the superior photocatalytic activity. Finally, thermogravimetric analysis (TGA) and derivative thermogravimetry (DTG) (Fig. S1) were employed to evaluate the thermal stability of the composite materials. While pure PV undergoes pronounced degradation above 300 °C, leaving a residual mass of approximately 60%, the *x*Bi-ZnO@PV heterojunctions exhibit markedly improved thermal stability, retaining about 90% residual mass at 800 °C. These findings confirm the high inorganic content within the composite and the protective spatial confinement provided by the cross-linked polymer network.^40^

**Fig. 3:**
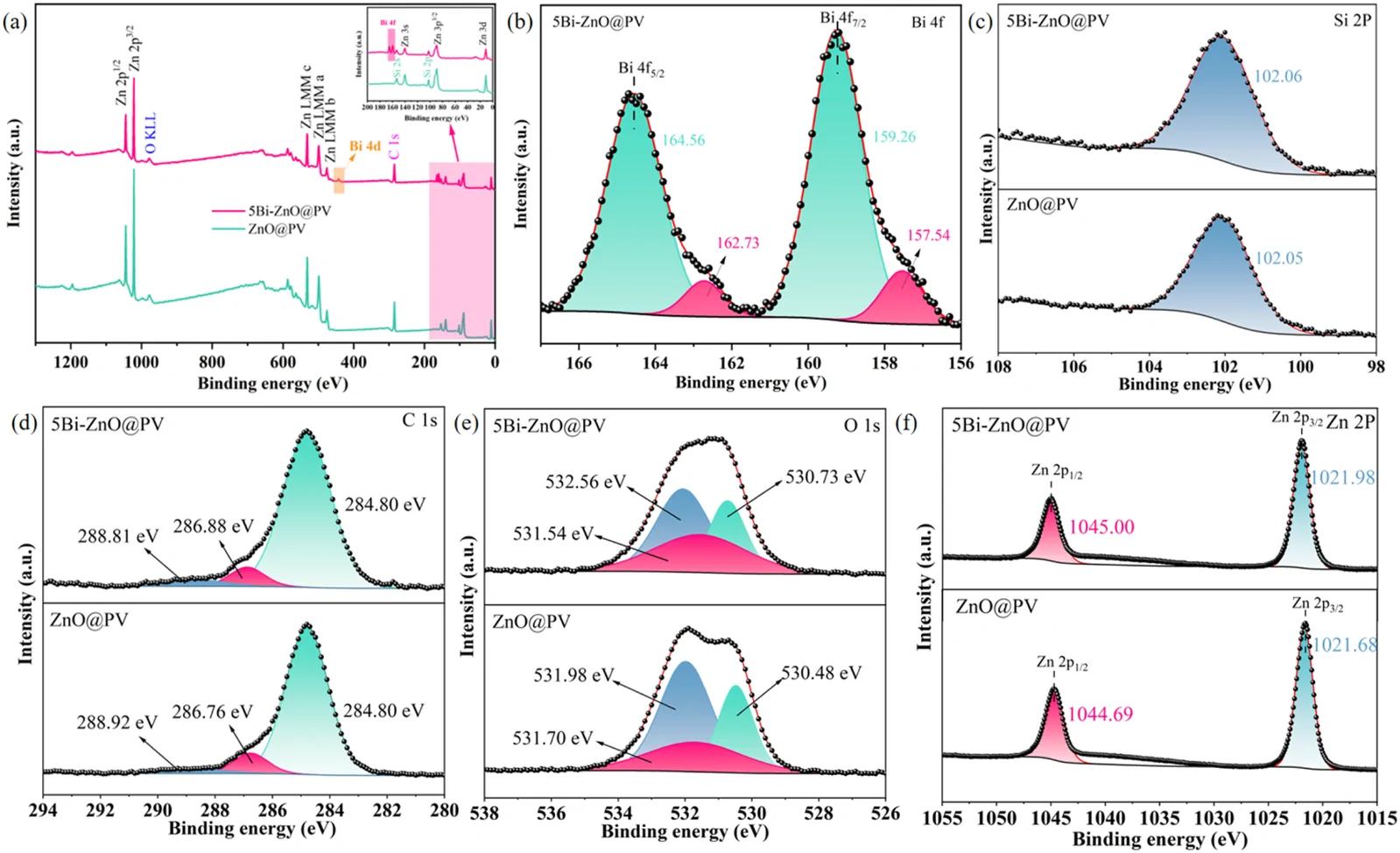
(a) XPS survey spectra of the ZnO@PV and 5Bi-ZnO@PV samples; (b) high-resolution XPS spectrum of Bi 4f for the 5Bi-ZnO@PV sample; (c) high-resolution XPS spectra of Si 2p for the ZnO@PV and 5Bi-ZnO@PV samples; (d) high-resolution XPS spectra of Zn 2p for the ZnO@PV and 5Bi-ZnO@PV samples; (e) high-resolution XPS spectra of C 1s for the ZnO@PV and 5Bi-ZnO@PV samples; (f) high-resolution XPS spectra of O 1s for the ZnO@PV and 5Bi-ZnO@PV samples.

### 3.3 Morphological evolution and interfacial assembly

The morphological evolution of the composites is dictated by the introduction of the bismuth precursor within the regulating PV matrix. Scanning electron microscopy (SEM) reveals that both the pure PV and the ZnO@PV samples exhibit a uniform morphology consisting of granular nanoparticles (Fig. 4a and b). However, the introduction of bismuth triggers a dramatic structural transformation (Fig. 4 and S2). As clearly observed in the 5Bi-ZnO@PV sample (Fig. 4c), prominent nanosheet-like structures, characteristic of Bi_2_O_2_CO_3_, spontaneously nucleate and intimately intertwine with the granular ZnO@PV network. EDS elemental mapping (Fig. 4d) further elucidates the spatial distribution and interfacial relationship of these phases. The Zn (purple) and Si (green) signals display synchronized distribution patterns, confirming that ZnO nanoparticles are homogeneously anchored within the PV matrix without macroscopic segregation. Notably, the Bi signal (orange) exhibits localized regions of high intensity that spatially correlate with the distinct nanosheet clusters observed in the SEM images. This spatial correspondence suggests that the Bi_2_O_2_CO_3_ nanosheets grow *in situ* using the ZnO@PV granules as nucleation templates. The overlapping of C, O, Si, Zn, and Bi signals throughout the architectures demonstrates the formation of an integrated heterostructure with intimate contact between the component phases.

**Fig. 4:**
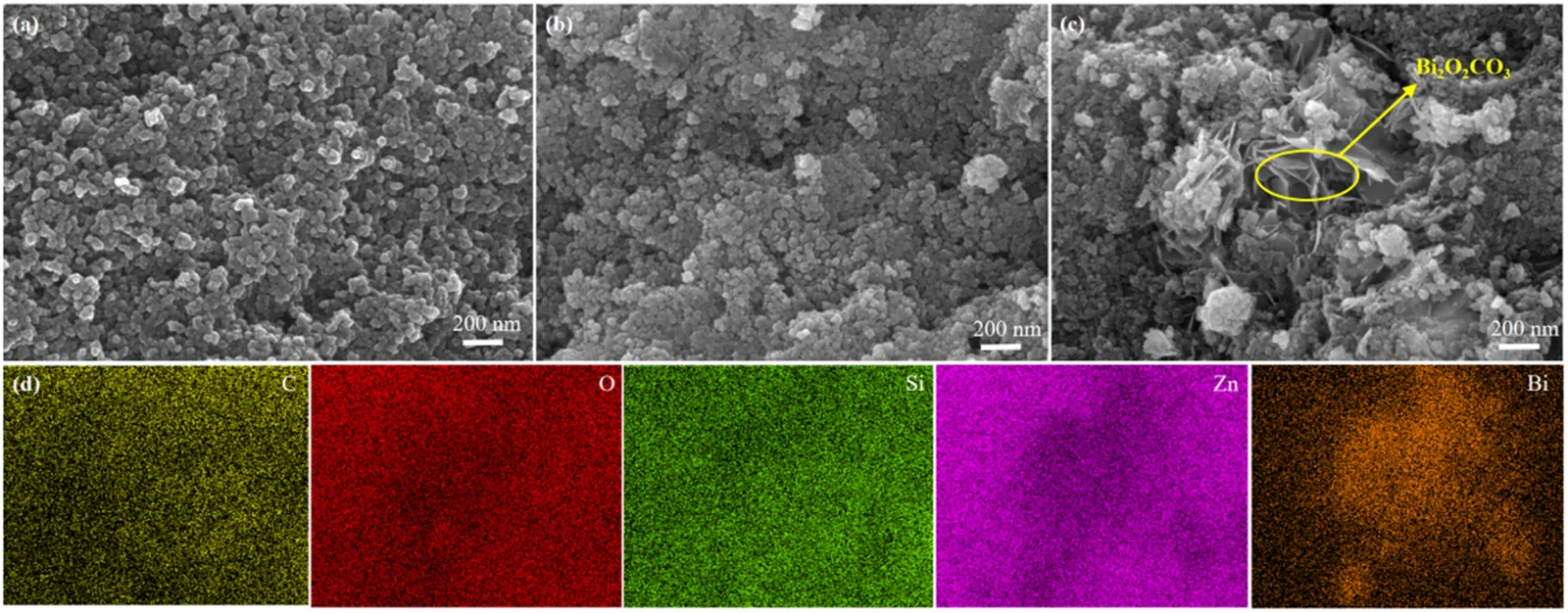
Morphological and elemental characterization. SEM images of the (a) pure PV, (b) ZnO@PV, and (c) 5Bi-ZnO@PV samples, highlighting the Bi_2_O_2_CO_3_ nanosheets. (d) Corresponding EDS elemental mapping images (C, O, Si, Zn, Bi) for the 5Bi-ZnO@PV composite.

To elucidate the nanoscale architecture and interfacial structure of the optimized 5Bi-ZnO@PV composite, TEM and HRTEM analyses were performed, as shown in Fig. 5. The TEM image in Fig. 5a reveals an elongated, dark-contrast domain embedded within and closely surrounded by a lighter granular matrix. Considering the higher mass-thickness contrast of the Bi-containing phase, together with the SEM morphology and EDS elemental mapping results, the dark nanosheet-like region is assigned to Bi_2_O_2_CO_3_, whereas the surrounding granular phase is predominantly associated with ZnO. The close spatial integration of the two components indicates that Bi_2_O_2_CO_3_ grows in intimate contact with the ZnO domains within the PV-derived framework, rather than forming an independently aggregated phase.

**Fig. 5:**
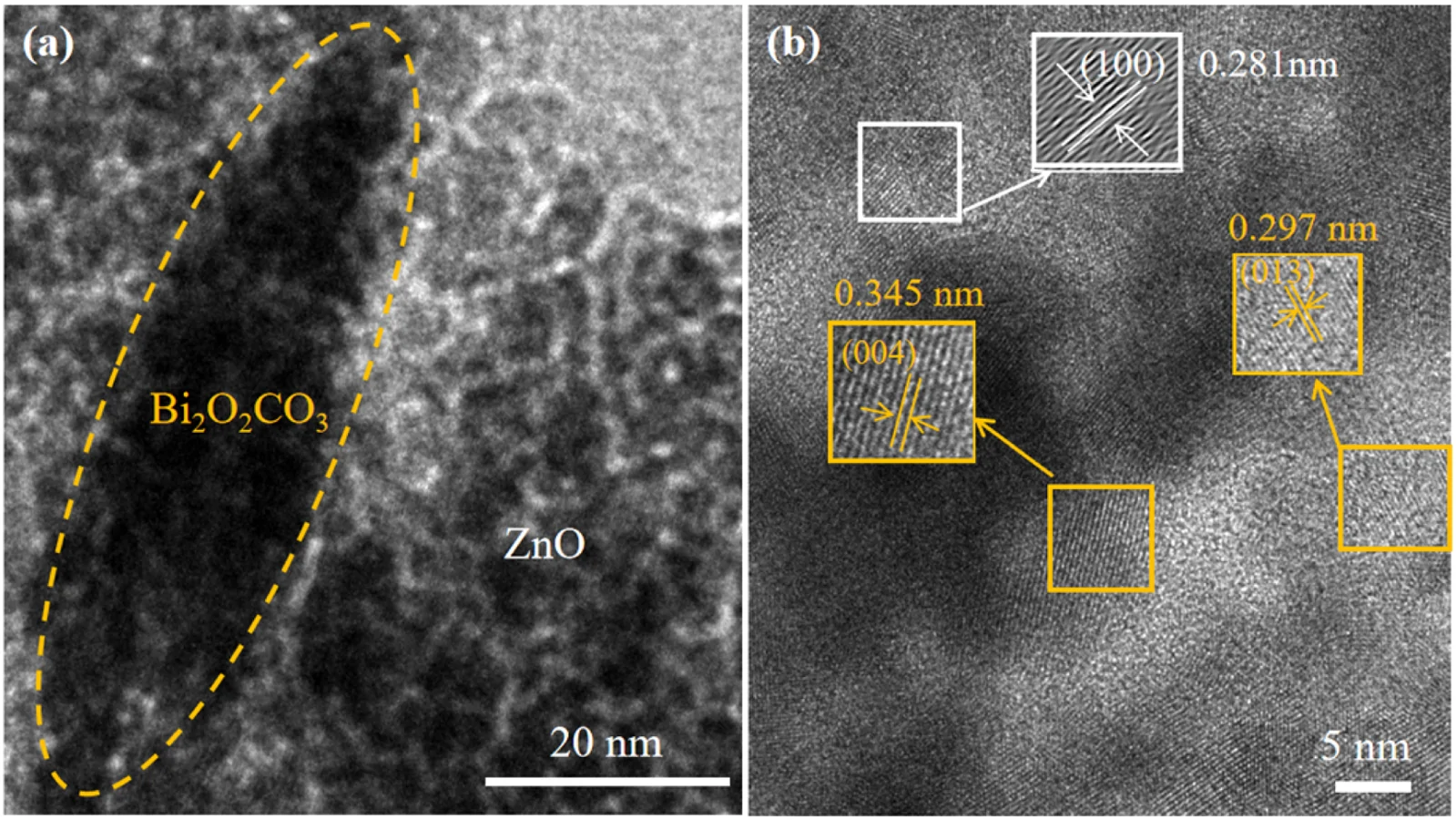
Nanoscale morphology and interfacial structure of the 5Bi-ZnO@PV composite. (a) TEM image showing a dark-contrast Bi_2_O_2_CO_3_ nanosheet-like domain embedded within the granular ZnO matrix. (b) HRTEM image displaying lattice fringes with interplanar spacings of approximately 0.281, 0.345, and 0.297 nm, corresponding to the ZnO (100), Bi_2_O_2_CO_3_ (004), and Bi_2_O_2_CO_3_ (013) planes, respectively.

The HRTEM image in Fig. 5b provides further crystallographic evidence for the coexistence of ZnO and Bi_2_O_2_CO_3_ at the nanoscale. Three sets of well-resolved lattice fringes are observed within adjacent crystalline regions. The measured interplanar spacing of approximately 0.281 nm is consistent with the (100) planes of hexagonal wurtzite ZnO, in agreement with the HRTEM results.^41^ The measured spacings of approximately 0.345 and 0.297 nm are consistent with the (004) and (013) planes of tetragonal Bi_2_O_2_CO_3_, respectively. These assignments are supported by the corresponding reference spacings of approximately 0.342 and 0.295 nm.^42^ Together with the XRD results, the lattice fringes observed in neighboring regions support the coexistence and close spatial association of ZnO and Bi_2_O_2_CO_3_ within the composite. Moreover, the close contact between the two crystalline phases indicates the establishment of abundant interfacial junctions within the composite. Such nanoscale interfacial coupling is expected to shorten the migration distance of photogenerated charge carriers and provide efficient pathways for interphase charge transfer. Meanwhile, the nanosheet-granule architecture increases the accessible interfacial area and suppresses excessive aggregation of the inorganic components within the PV-derived matrix. These structural characteristics provide a favorable physical basis for the enhanced carrier separation and reduced electron–hole recombination observed in the subsequent optical and photoelectrochemical analyses.^43–45^

Furthermore, N_2_ adsorption–desorption analysis was conducted to elucidate the evolution of the specific surface area and porous structure (Fig. S5). As shown in Fig. S5a, all three samples display typical Type IV isotherms with noticeable hysteresis loops at higher relative pressures (*P*/*P*_0_ > 0.4), indicating the inherent mesoporous nature of the materials. Their BET specific surface areas are 432.4, 208.5, and 372.5 m^2^ g^−1^, respectively. The corresponding adsorption-derived pore volumes, evaluated at *P*/*P*_0_ = 0.99, are 1.01, 0.26, and 0.39 cm^3^ g^−1^. The pure PV substrate exhibits the highest N_2_ adsorption capacity. However, upon the loading of dense granular ZnO nanoparticles (ZnO@PV), the overall adsorption capacity decreases significantly, implying that the ZnO nanoparticles severely block or fill the intrinsic pores of the PV matrix. Remarkably, the *in situ* growth of the Bi_2_O_2_CO_3_ phase in the 5Bi-ZnO@PV composite leads to a distinct recovery in adsorption capacity. This structural optimization is further verified by the corresponding Barrett–Joyner–Halenda (BJH) pore size distribution curves (Fig. S5b). While the pores in the ZnO@PV sample are severely suppressed, the 5Bi-ZnO@PV composite exhibits a broader and much more pronounced pore volume distribution centered in the mesoporous range (2–20 nm). This vital recovery of porosity is primarily attributed to the hierarchical interlacing of the Bi_2_O_2_CO_3_ nanosheets, which acts as a structural scaffold to effectively mitigate the severe agglomeration and restacking of the underlying granular nanoparticles, thereby generating abundant slit-like interfacial mesopores. Ultimately, this rationally designed hierarchical mesoporous architecture not only guarantees an abundance of exposed catalytic active sites but also facilitates the rapid mass diffusion of targeted molecules, establishing a solid structural foundation for superior catalytic performance.

Remarkably, the construction of the Bi_2_O_2_CO_3_ heterojunction in the 5Bi-ZnO@PV composite triggers a dramatic transition to a hydrophilic state. Although the initial droplet exhibits a relatively high contact angle upon impact (0 s), it undergoes rapid and continuous spreading. The WCA decreases sharply from ∼110.6° at 1.5 s, to ∼51.6° at 22.6 s, down to ∼25.0° at 24.4 s, and eventually achieves near-complete wetting (superhydrophilicity) by 29.3 s. This outstanding dynamic wetting behavior is the result of a strong synergistic effect between the chemical composition and the topological structure. Chemically, polar oxygen-containing groups at the composite surface may contribute to interactions with water molecules. Together with the hierarchical surface morphology and pore structure, these surface chemical environments may account for the observed wetting behavior. Topologically, the rationally designed hierarchical mesoporous architecture, constructed by the interlaced 2D Bi_2_O_2_CO_3_ nanosheets (as observed in SEM and BET), induces a potent capillary effect within the slit-like pores. According to Wenzel's wetting model, this hierarchical roughness amplifies the inherent hydrophilicity, forcefully drawing the water droplet into the internal porous network.^46^ Consequently, this superhydrophilic nature guarantees that targeted aqueous reactant molecules can rapidly diffuse into the deeper mesopores, achieving intimate contact with the seamlessly coupled ZnO/Bi_2_O_2_CO_3_ interfaces and maximizing the utilization efficiency of the photo-generated charge carriers (Fig. 6).

**Fig. 6:**
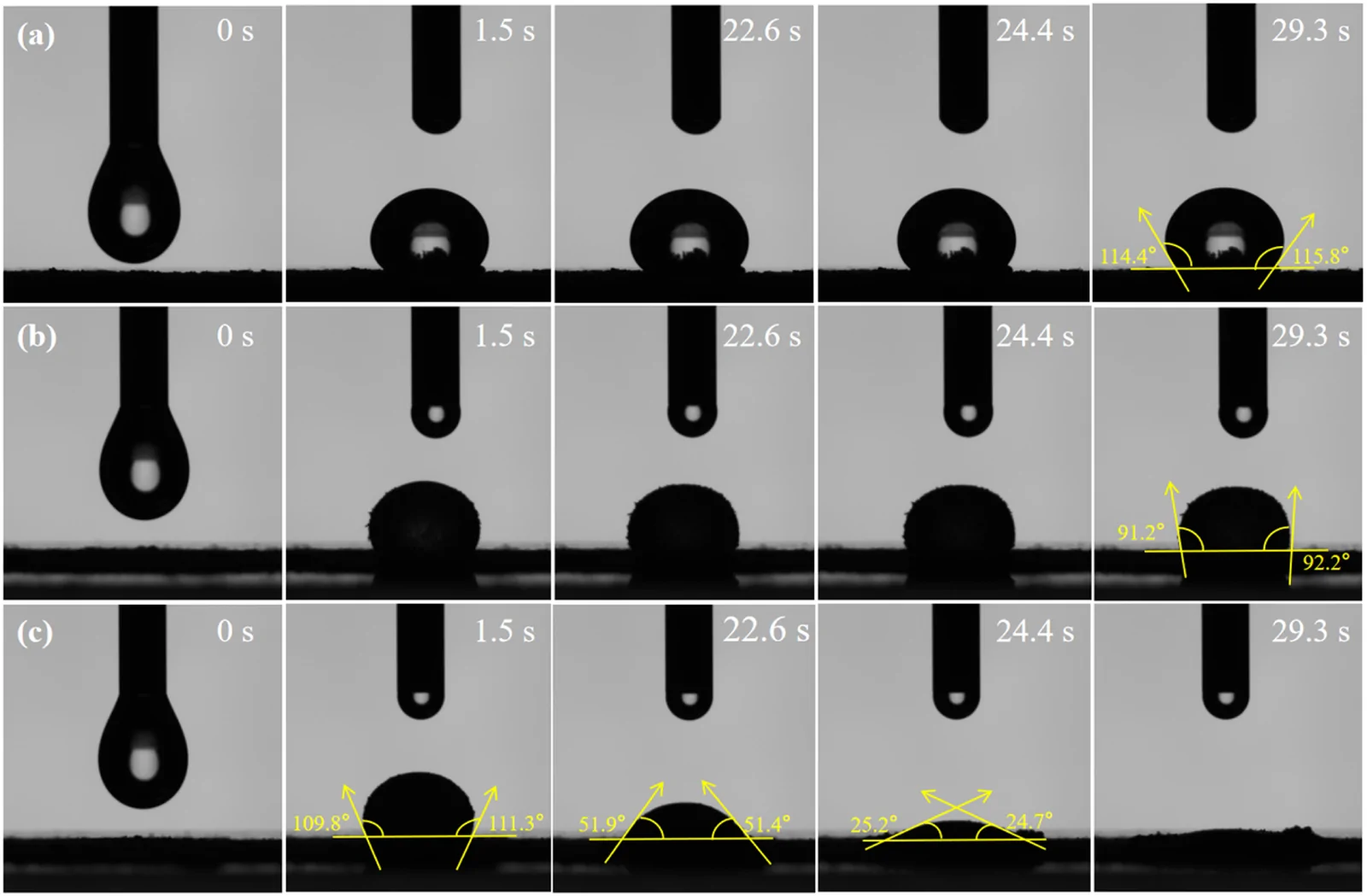
Time-dependent WCA measurements of (a) the pure PV, (b) ZnO@PV, and (c) 5Bi-ZnO@PV samples, demonstrating the dynamic transition from hydrophobicity to exceptional hydrophilicity.

### 3.4 Photocatalytic activity evaluation

The photocatalytic activity of the synthesized samples was systematically evaluated through the degradation of MO under blue light (*λ*_max_ = 450 nm, 50 W) irradiation. Initially, the optimal inorganic loading ratio within the polymer matrix was established (Fig. 7a). The ZnO@PV sample exhibited the most favorable degradation efficiency, striking an optimal balance between active site density and reactant diffusion; thus, this ratio was adopted for further structural modification. Building upon this optimized matrix, the profound effect of the Bi_2_O_2_CO_3_ integration was investigated (Fig. 7b). Control experiments revealed that the pristine PV matrix is practically inert under illumination. During the 30 min initial dark phase, the functionalized composites demonstrated substantial adsorption capacities (approximately 40–50%), a property directly attributable to the hierarchical mesoporous architecture identified in the BET analysis. Upon illumination, the synergistic advantages of the heterostructure became starkly apparent. The 5Bi-ZnO@PV composite displayed the highest degradation activity, achieving near-complete removal of MO (92.6%) within 40 min. In contrast, the physical powder mixture (5Bi-ZnO(1.0)) exhibited negligible adsorption in the dark and markedly inhibited photodegradation kinetics, underscoring the pivotal role of the PV matrix in preconcentrating reactant molecules and suppressing extensive nanoparticle agglomeration. It is also noteworthy that overdoping (7Bi sample) led to a slight diminution in efficiency, likely because excessive Bi_2_O_2_CO_3_ nanosheets may act as recombination centers for charge carriers or induce a light-shielding effect.^47,48^ The reaction kinetics of the MO degradation were quantitatively analyzed using the pseudo-first-order kinetic model, expressed as −ln(*C*_*t*_/*C*_0_) = *kt* (Fig. 7c). The optimal 5Bi-ZnO@PV composite achieved a remarkable apparent reaction rate constant (*k*) of 0.0519 min^−1^, which is approximately 7.3 times higher than that of the bare ZnO@PV counterpart (0.0071 min^−1^). This dramatic kinetic enhancement unambiguously validates the superiority of the newly constructed ZnO/Bi_2_O_2_CO_3_ heterojunction in accelerating charge separation and transfer. Furthermore, the effect of catalyst dosage on MO removal was optimized (Fig. 7d). The results indicate that increasing the mass loading progressively maximizes the synergistic interplay between surface dark adsorption and subsequent photodegradation. An optimal dosage of 3 g L^−1^ was identified, beyond which the total removal rate plateaus near 100%, likely due to the limited light penetration and increased scattering effect in a concentrated suspension. To evaluate the practical applicability of the catalyst under diverse environmental conditions, the impact of initial solution pH on MO degradation was meticulously investigated (Fig. 7e). The composite exhibits superior performance in acidic to moderately slightly acidic environments (pH 2–6), characterized by dark adsorption (∼60% at pH 2) and rapid photodegradation. Conversely, under alkaline conditions (pH 8–10), the degradation efficiency noticeably declines. This pH-dependent behavior is primarily governed by the electrostatic interactions between the catalyst surface and the dye molecules.^49^ At lower pH values, the surface of the composite becomes protonated and positively charged, which establishes a strong electrostatic attraction with the anionic MO molecules, thereby facilitating intimate contact and rapid mass transfer. In alkaline solutions, both the catalyst surface and MO molecules carry negative charges, inducing strong electrostatic repulsion that significantly hinders the adsorption of MO onto the active catalytic sites. Furthermore, authentic industrial wastewater typically contains various inorganic salts that can potentially interfere with the catalytic process. Thus, the impact of typical co-existing anions (SO_4_^2−^, CO_3_^2−^ and Cl^−^) was examined (Fig. 7f). The presence of SO_4_^2−^, and Cl^−^ exerts only a negligible to mild inhibitory effect on the overall degradation efficiency. However, the introduction of CO_3_^2−^ suppresses both the initial dark adsorption and the subsequent photoactivity. This pronounced inhibition is dual-faceted: firstly, CO_3_^2−^ undergoes hydrolysis to increase the solution pH, which is thermodynamically unfavorable for anionic MO adsorption as demonstrated above; secondly, carbonate ions are well-documented scavengers for reactive oxygen species (ROS), particularly reactive hydroxyl radicals (˙OH) and photogenerated holes (h^+^). The quenching of these crucial radicals directly consumes the primary driving forces for MO degradation. Despite these extreme interferences, the 5Bi-ZnO@PV composite still maintains substantial catalytic viability, underscoring its excellent structural stability and high potential for practical wastewater remediation applications.

**Fig. 7:**
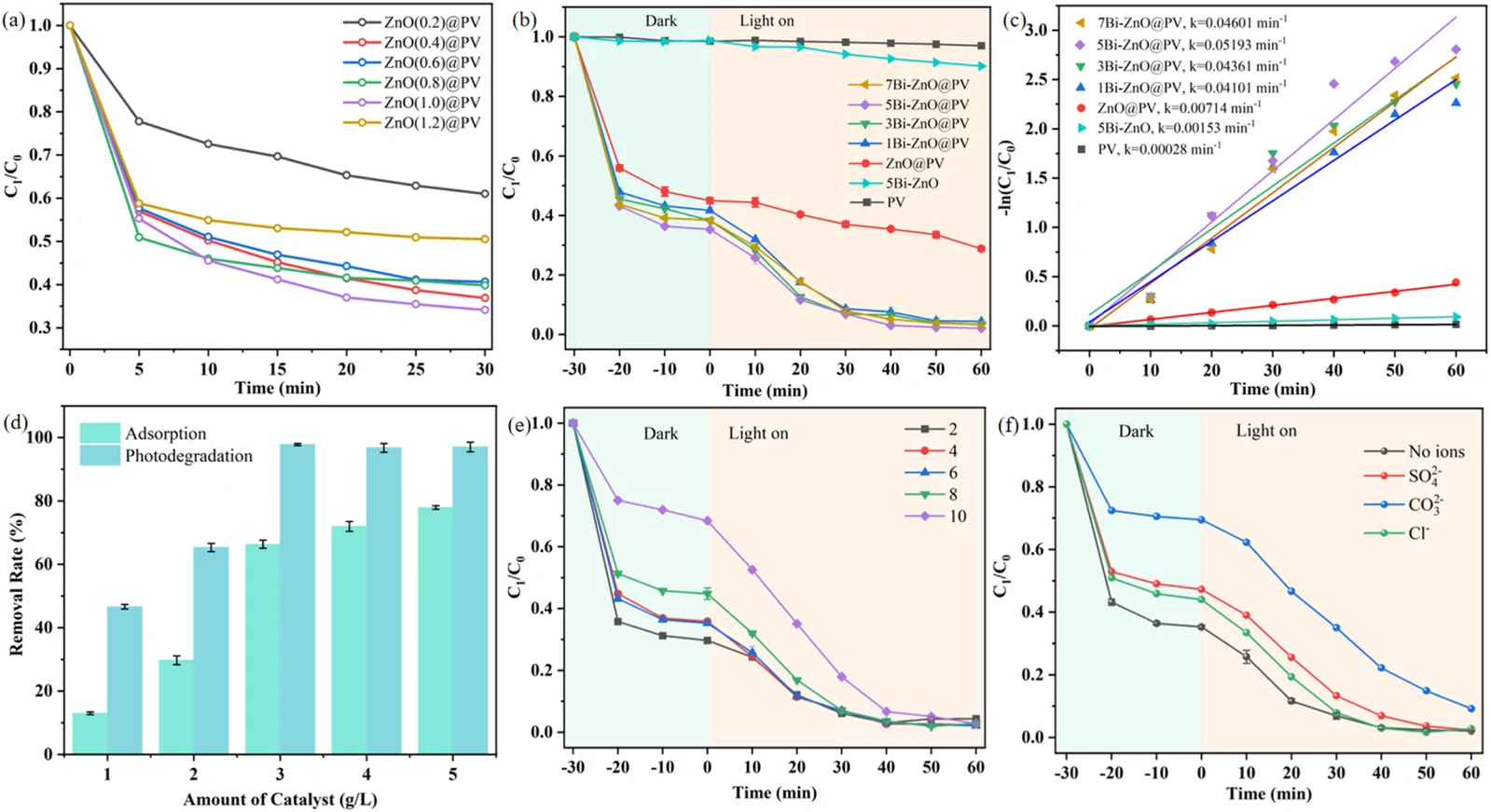
Photocatalytic performance evaluation. (a) Optimization of the ZnO precursor content for MO degradation; (b) photocatalytic degradation curves of MO over the pristine PV, 5Bi-ZnO(1.0) powder, ZnO@PV, and various *x*Bi-ZnO@PV composites under 450 nm blue LED irradiation; (c) corresponding pseudo-first-order kinetic linear fitting curves; (d) effect of catalyst dosage on the adsorption and total removal rates of MO; (e) effect of initial solution pH on the degradation efficiency of the 5Bi-ZnO@PV composite; (f) effect of typical co-existing anions (0.01 mol L^−1^ of SO_4_^2−^, CO_3_^2−^, and Cl^−^) on the photocatalytic performance.

To verify the practical applicability of the optimally designed catalyst, the 5Bi-ZnO@PV composite was initially tested against a broad spectrum of individual recalcitrant water pollutants (Fig. 8a). Impressively, the catalyst maintained excellent degradation efficiency across diverse targets, effectively mineralizing both traditional dyes (*e.g.*, RhB, MB) and persistent organic pollutants (*e.g.*, LVX, BPA), demonstrating strong universality. More importantly, to simulate realistic and complex wastewater conditions, a competitive degradation experiment using a binary mixture of MO (6.5 ppm) and RhB (3.4 ppm) was conducted (Fig. 8b). The time-dependent UV-vis absorption spectra display two distinct initial peaks corresponding to the characteristic absorptions of MO (∼464 nm) and RhB (∼554 nm). Upon light irradiation, both absorption peaks diminished synchronously and rapidly. This remarkable simultaneous degradation capability indicates that the hierarchical 5Bi-ZnO@PV composite possesses exceptional non-selective advanced oxidation capabilities and is resistant to the competitive adsorption of mixed complex molecules. This outstanding performance in a multi-component system provides compelling evidence for its immense potential in actual environmental remediation.

**Fig. 8:**
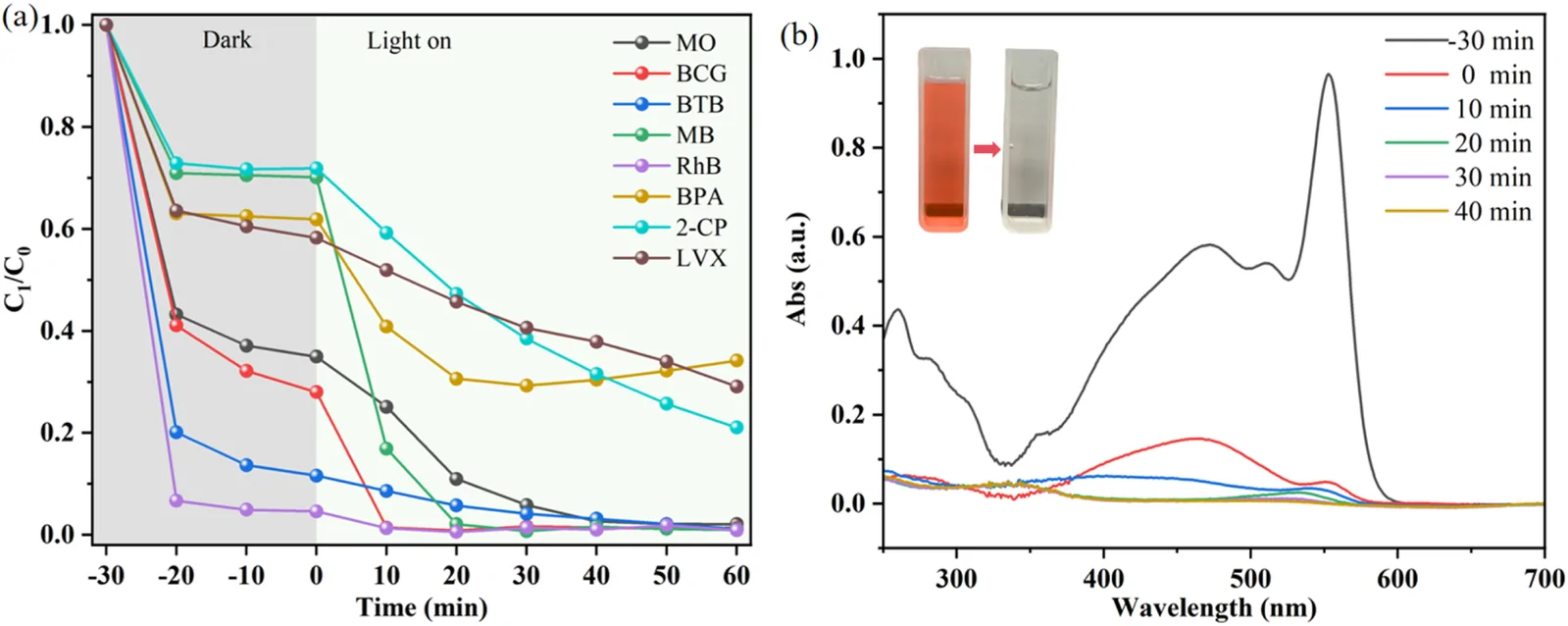
(a) Photocatalytic degradation efficiencies of the optimal 5Bi-ZnO@PV composite against various representative organic pollutants (dyes and antibiotics); (b) time-dependent UV-vis absorption spectra for the simultaneous degradation of a binary mixture containing MO (6.5 ppm) and RhB (3.4 ppm) over the 5Bi-ZnO@PV composite.

### 3.5 Optical properties and charge transfer dynamics

To elucidate the fundamental origin of the enhanced photocatalytic performance, the optical absorption properties and charge carrier dynamics of the composites were systematically investigated. The DRS (Fig. 9a) demonstrate that the bare ZnO@PV sample predominantly absorbs light in the ultraviolet (UV) region. However, the introduction of the Bi_2_O_2_CO_3_ phase induced a substantial red-shift of the absorption edge. Notably, the Bi-incorporated composites displayed an extended absorption shoulder in the visible light region (highlighted by the dashed box in Fig. 9a), enhancing visible light utilization. Consequently, the corresponding Tauc plots (Fig. 9b) reveal that the calculated bandgap energy exhibits a tailored reduction compared to the pristine ZnO composite, facilitating an easier excitation of electrons under light irradiation. The separation, migration, and recombination efficiencies of the photogenerated electron–hole pairs were scrutinized using steady-state photoluminescence (PL) alongside photoelectrochemical evaluations.^50^ In the PL spectra (Fig. 9c), the ZnO@PV sample displayed a prominent, broad emission peak centered around 530 nm, which is typically ascribed to the radiative recombination of charge carriers mediated by intrinsic defects within the ZnO lattice. Upon Bi integration, this emission intensity is drastically quenched, reaching its absolute minimum in the optimally loaded 5Bi-ZnO@PV sample. This pronounced quenching provides compelling optical evidence that the rationally constructed ZnO/Bi_2_O_2_CO_3_ heterojunction creates an efficient interfacial transfer pathway for photogenerated electrons, thereby profoundly suppressing their wasteful radiative recombination. This underlying mechanism is strongly corroborated by the photoelectrochemical measurements. As illustrated in the transient photocurrent responses (Fig. 9d), the 5Bi-ZnO@PV electrode exhibited a remarkably enhanced and stable photocurrent density over multiple light on–off cycles compared to its ZnO@PV counterpart. This signifies a massive and rapid generation of mobile charges, coupled with superior interfacial separation efficiency. Furthermore, EIS (Fig. 9e) reveals a smaller Nyquist arc radius for the 5Bi-ZnO@PV sample. This confirms a drastically reduced interfacial charge transfer resistance, ensuring that the spatially separated electrons and holes can smoothly migrate to the catalyst surface to participate in the targeted redox degradation reactions.

**Fig. 9:**
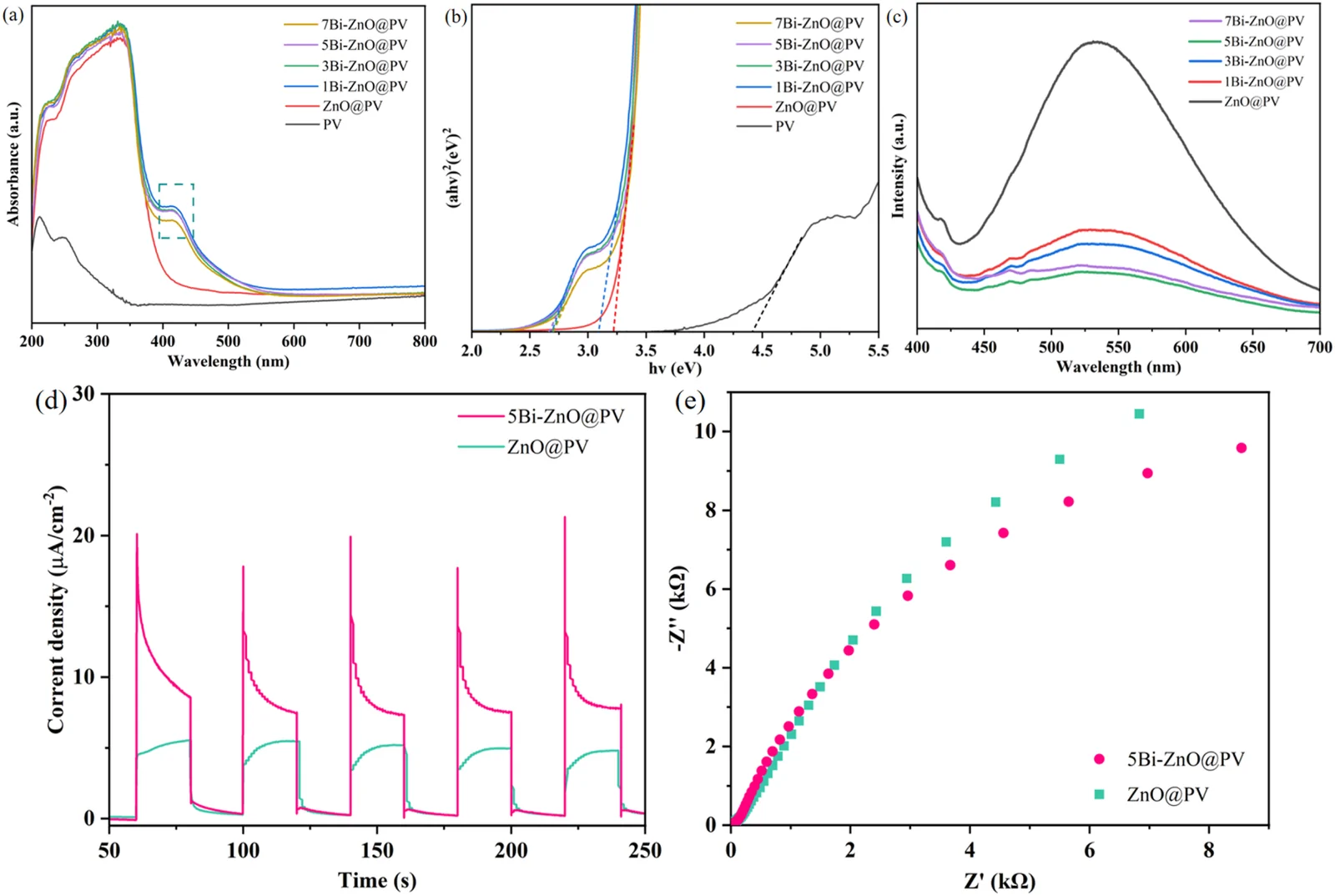
Optoelectronic properties and charge transfer dynamics. (a) DRS and (b) the corresponding Tauc plots for bandgap calculation of the pure PV, ZnO@PV, and various *x*Bi-ZnO@PV composites. (c) Steady-state photoluminescence (PL) spectra. (d) Transient photocurrent responses and (e) EIS Nyquist plots of the ZnO@PV and optimized 5Bi-ZnO@PV electrodes.

### 3.6 Photocatalytic mechanism and reusability

To unmask the primary reactive species governing the photocatalytic process, systematic radical trapping experiments were performed (Fig. 10a). The introduction of *p*-BQ (a scavenger for ˙O^2−^) and ammonium oxalate (AO, a scavenger for h^+^) severely inhibited the degradation efficiency, identifying ˙O^2−^ and photogenerated h^+^ as the dominant active species driving the degradation. In contrast, isopropanol (IPA, a scavenger for ˙OH) caused only minor inhibition, indicating a secondary role for ˙OH. Interestingly, the addition of K_2_S_2_O_8_ (an efficient electron scavenger) drastically accelerated the reaction, reaching near-complete degradation within 10 min. This provides indirect yet powerful evidence that the rapid extraction of photogenerated electrons from the catalyst surface effectively suppresses charge recombination, thereby liberating more active species for surface oxidation.^51^ The generation of these reactive oxygen species (ROS) was conclusively verified *via in situ* EPR spin-trapping (Fig. 10b). While no signals were detected in the dark, intense characteristic quartet signals for DMPO (5,5-dimethyl-1-pyrroline *N*-oxide)-˙O^2−^ and DMPO^−^˙OH emerged immediately upon light irradiation. These results provide direct evidence that the 5Bi-ZnO@PV heterojunction possesses sufficient redox potential to trigger the production of ROS. Based on the integrated structural, optical, and photoelectrochemical evidence, the superior activity stems from the construction of an intimate, defect-minimized interfacial contact between the ZnO granules and Bi_2_O_2_CO_3_ nanosheets. This 2D/3D hierarchical architecture not only provides abundant accessible active sites but also functions as an express channel for rapid interfacial charge migration, significantly prolonging carrier lifetimes.

**Fig. 10:**
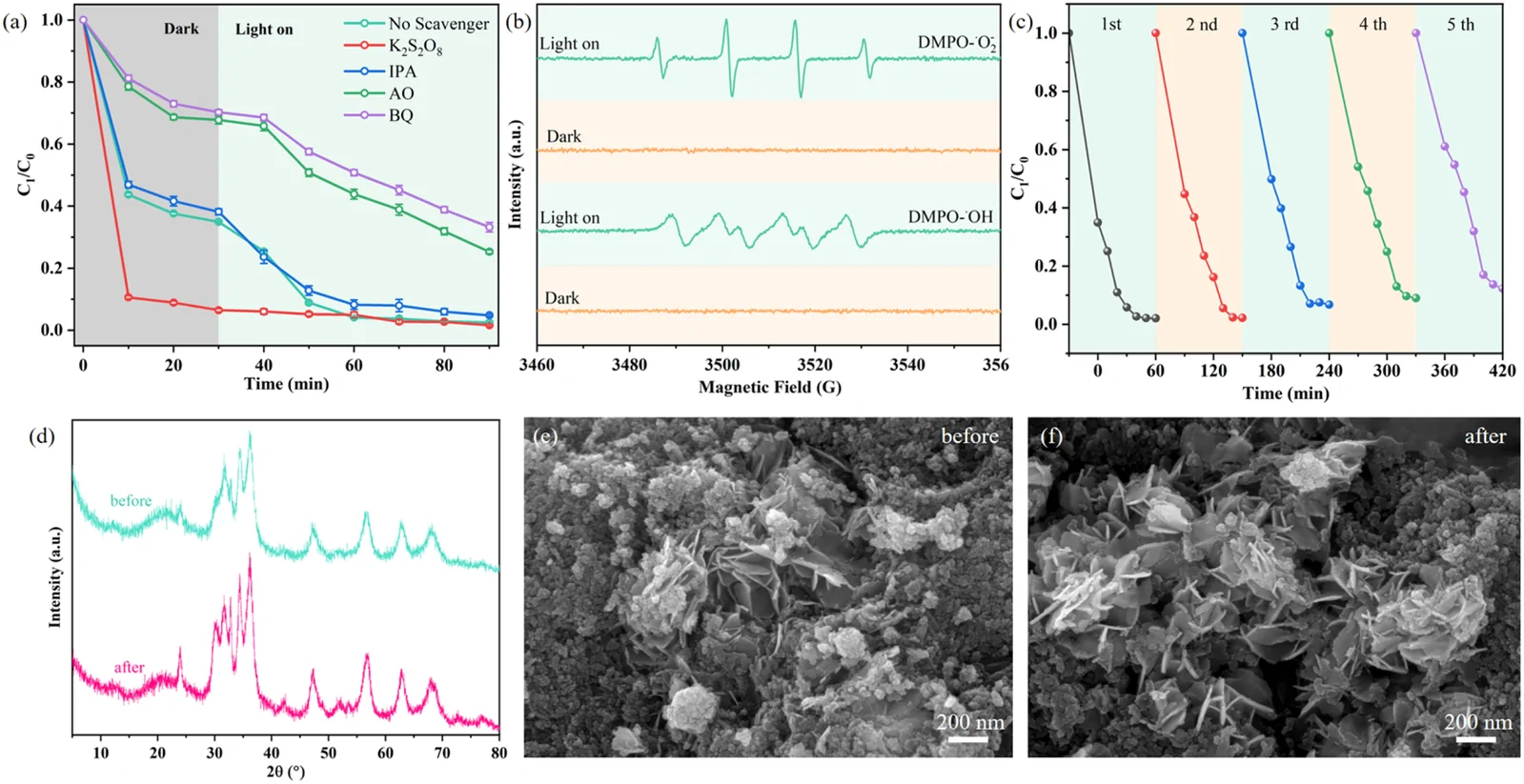
Investigation of photocatalytic mechanism and stability. (a) Radical trapping experiments for MO degradation over the 5Bi-ZnO@PV composite using various scavengers. (b) EPR spectra of DMPO^−^˙O^2−^ and DMPO^−^˙OH recorded over the 5Bi-ZnO@PV composite under dark and light conditions. (c) Reusability of the 5Bi-ZnO@PV catalyst over five consecutive cycles. Comparison of (d) XRD patterns and SEM images (e) before and (f) after five photocatalytic cycles.

Furthermore, the VB-XPS spectrum of 5Bi-ZnO@PV shows an apparent valence-band onset at approximately 2.59 eV on the C 1s-corrected binding-energy scale (Fig. S6a), as determined by linear extrapolation of the leading edge to the background. The UPS spectrum exhibits a secondary-electron cutoff at approximately 23.53 eV on the recorded energy scale (Fig. S6b), acquired using He I radiation (21.22 eV) with a sample bias of −5 V. The apparent valence-band onset provides an experimental reference for the occupied states at the composite surface. The photoelectrochemical measurements support improved charge-transfer behavior, while the scavenging and EPR results support the participation of reactive species in pollutant degradation.^52^ Therefore, a tentative direct S-scheme charge transfer pathway is proposed to elucidate the enhanced photocatalytic performance. Given the differences in work functions (*Φ*) and Fermi levels (*E*_f_) between the two semiconductors, electrons in ZnO (possessing a higher *E*_f_) spontaneously migrate across the intimate interface toward Bi_2_O_2_CO_3_ under dark conditions until Fermi equilibrium is reached. This spontaneous charge redistribution induces interfacial polarization, with negative charges accumulating on the Bi_2_O_2_CO_3_ side and positive charges on the ZnO side, thereby establishing an internal electric field (IEF) directed from ZnO to Bi_2_O_2_CO_3_. Simultaneously, the energy bands of ZnO bend upward, while those of Bi_2_O_2_CO_3_ bend downward at the interface. Under simulated solar illumination, electrons in the valence bands (VBs) of both ZnO and Bi_2_O_2_CO_3_ are excited to their respective conduction bands (CBs). Theoretically, this staggered band configuration could follow either a traditional Type-II or an S-scheme mechanism. In a Type-II model, the IEF would drive electrons from the CB of Bi_2_O_2_CO_3_ to that of ZnO, and holes from the VB of ZnO to that of Bi_2_O_2_CO_3_. However, this would result in the accumulation of carriers on potentials with weaker redox capacity, which contradicts our observation of the simultaneous generation of high-potential ˙O^2−^ and h^+^ species. Therefore, an S-scheme mechanism is more plausible. In this pathway, the synergistic effect of the IEF, band bending, and coulombic attraction facilitates the recombination of the relatively useless electrons from the CB of Bi_2_O_2_CO_3_ with the holes from the VB of ZnO at the interface. This selectively preserves the photogenerated electrons with strong reduction potential in the CB of ZnO (ECB ≈ −0.31 eV *vs.* NHE) to reduce O_2_ into ˙O^2−^, and the holes with powerful oxidation capacity in the VB of Bi_2_O_2_CO_3_ (EVB ≈ 2.39 eV *vs.* NHE) to directly oxidize organic pollutants. These spatially separated, active charge carriers subsequently migrate to the superhydrophilic catalyst surface, driving the efficient degradation of MO and other recalcitrant molecules.

Beyond catalytic activity, the long-term stability and reusability of a photocatalyst are paramount for practical applications. During five consecutive photocatalytic cycles (Fig. 10c), the final MO removal efficiency gradually decreased to 79.9%. The composite therefore retained substantial photocatalytic activity, although a moderate activity loss was observed. This decline may be associated with unavoidable catalyst loss during recovery, adsorption of persistent reaction intermediates on active sites, and partial evolution of the surface structure during repeated washing and drying. The recycled catalyst retained the characteristic diffraction peaks of ZnO and Bi_2_O_2_CO_3_, but noticeable variations in their relative intensities were observed (Fig. 10d). These variations suggest changes in preferred orientation, relative crystallinity, or surface exposure of the lamellar Bi_2_O_2_CO_3_ domains rather than complete preservation of the initial structure. Table 1 presents a performance comparison between 5Bi-ZnO@PV and other recent photocatalytic systems. Our hierarchical heterojunction reached 92.6% MO degradation in 40 min using a 450 nm light-emitting diode (LED), outperforming the PVDF/CuFe/LDH membrane (79%) and Ag/AgI/Bi_3_O_4_Cl (61%) in both speed and total efficiency. The composite prepared through sol–gel processing followed by low-temperature aging at 80 °C retained an MO removal efficiency of 79.9% after five photocatalytic cycles. These results underscore the synergistic effect of the Bi-ZnO/PV architecture, providing an optimal trade-off between rapid degradation kinetics and long-term catalytic endurance.

**Table 1:** Comparative evaluation of the 5Bi-ZnO@PV composite with recently reported photocatalytic systems for the degradation of MO under various irradiation conditions

Photocatalytic systems	Preparation process and steps (temperature)	Irradiation source/Wavelength/Power	Time (min)	Degradation rate (%)	Reusability	Ref.
Ag/ZnO composite	Two-step template-assisted calcination (500 °C)	Hg lamp/UV light/250 W	120	∼100	After 6 cycles: ∼100%	53
ZnCuP_2_O_7_/TiO_2_ composite	Two-step solid-state reaction (600 °C)	UV lamps/254 nm/18 W	300	97.21	—	54
ZnO@SnIn_4_S_8_ core–shell nanorods	Three-step wet-chemical synthesis (300 °C)	Solar simulator/AM 1.5/100 mW cm^−2^	90	99.8	After 9 cycles: 93.5%	55
WO_3_/SnSe composite	Three-step hydrothermal method (100 °C)	Visible light/—/—	60	99.71	After 5 cycles: 86.46%	56
Sn_3_O_4_/In_2_O_3_ heterostructure	Three-step MOF-template calcination and solvothermal (180 °C)	Xenon lamp/—/500 W	40	90.6	After 4 cycles: 80.6%	57
Sc/ZnO@SiO_2_	Three-step co-precipitation and plasma treatment (200 °C)	Xenon lamp/visible light/500 W	25	∼100	After 5 cycles: 98%	58
Fe_2_O_3_/g-C_3_N_4_ nanocomposite	Two-step calcination	OSRAM lamp/visible light/150 W	180	49	—	59
	Precipitation (550 °C)					
g-C_3_N_4_/NIPAM-DADMAC	Two-step template-assisted calcination (500 °C)	Xenon lamp/visible light/100W	120	98.15	After 8 cycles: 77.92%	60
EP/XG/Ag@AgCl porous hydrogel spheres	CO_2_ pressure-decompression & dripping (60 °C)	Xenon lamp/400 nm/300 W	20	99	—	61
Ag/AgCl/cordierite composite	Three-step method: deposition, precipitation, and photoreduction (30–60 °C)	Xenon lamp/420 nm/300 W	25	∼100	After 5 cycles: 88.50%	62
CuO/g-C_3_N_4_ heterojunction	Three-step: hydrothermal (120 °C), calcination (550 °C), interfacial calcination (300 °C)	Xenon lamp/420 nm/300 W	40	98.4	After 5 cycles: 98.1%	63
ZnO/TiO_2_	Two-step sol–gel calcination (450 °C)	Xenon lamp/300–2500 nm/300 W	80	∼100	—	64
Au/g-C_3_N_4_ composite	Two-step calcination impregnation method (550 °C)	LED lamp/420 nm–700 nm/68 mW cm^−2^	180	98.9	—	65
PVDF/CuFe/LDH membrane	Two-step coprecipitation	UV-LED lamp/394 nm/—	45	79	—	66
	Phase inversion method (65 °C)					
Ag/AgI/Bi_3_O_4_Cl	Hydrothermal precipitation (220 °C)	LED lamp/visible light/80 mW cm^−2^	180	61	After 4 cycles: 61%	67
BiVO_4_/ZnIn_2_S_4_ composite	One-step hydrothermal method (160 °C)	LED lamp/400–780 nm/30 W	240	86	After 4 cycles: 86%	68
5Bi-ZnO@PV composite	One-pot sol–gel followed by low-temperature aging (80 °C)	LED lamp/450 nm/50 W	40	92.6	After 5 cycles: 79.9%	This work

## 4. Conclusion

In summary, this work demonstrates that a reactive PVMDMS-derived network can actively regulate inorganic phase formation and heterointerface assembly under mild processing conditions. During sol–gel processing and subsequent aging at 80 °C, hydrolysis and condensation of PVMDMS generate a cross-linked siloxane environment that restricts extensive aggregation and promotes the spatial integration of Bi_2_O_2_CO_3_ nanosheets with granular ZnO domains. This polymer-regulated assembly produces a hierarchical architecture with accessible mesoporosity and intimate nanoscale heterointerfacial contact, which is correlated with improved light utilization, suppressed charge-carrier recombination, and accelerated interfacial charge transport. As a functional consequence, the optimized 5Bi-ZnO@PV composite exhibits an apparent methyl orange degradation rate constant of 0.0519 min^−1^, approximately 7.3 times that of ZnO@PV. Scavenger and EPR analyses identify ˙O_2_^−^ and h^+^ as the dominant reactive species, whereas the detailed S-scheme-like charge-transfer pathway remains a proposed interpretation pending more direct evidence of band bending and work-function differences. The composite retains 79.9% removal efficiency after five cycles, although partial structural evolution occurs during reuse. More broadly, these results establish a structure–function relationship in which reactive polymer confinement governs inorganic hierarchical organization and heterointerfacial coupling, providing a general materials-design concept for polymer-regulated functional hybrid systems.

## Conflicts of interest

The authors declare no conflict of interest.

## Supplementary Material

RA-OLF-D6RA08383H-s001

## Data Availability

All data generated or analyzed during this study are included in this published article (and its supplementary information (SI) files). Supplementary information is available. See DOI: https://doi.org/10.1039/d6ra08383h.
