# Supplementary material for: Reactive polymer confinement enables low-temperature Bi2O2CO3/ZnO heterojunction assembly for visible-light photocatalysis
Source: RSC Adv. 2026 Nov 9. Online ahead of print. doi: 10.1039/d6ra08383h (PMC13746121; doi:10.1039/d6ra08383h)
Supplement: Supplementary file 1 [file RA-OLF-D6RA08383H-s001.pdf]

Supplementary Information (SI) for RSC Advances.
This journal is © The Royal Society of Chemistry

Supporting Information

Reactive Polymer Confinement Enables Low-Temperature Bi₂O₂CO₃/ZnO Heterojunction Assembly for Visible-Light Photocatalysis

Xiangfei Chen,[a] Yinji Lu,[a] Yi Jin,[b] Jie Li,[b] Wentao Zhu,[b] Yanwen Lv,[a,b,] Jiangang Yu[b,*]*

[a]Department of Chemical and Material Engineering, Quzhou College of Technology, No. 18 Jiangyuan Road, Quzhou 324002, Zhejiang, China

[b]College of Chemical and Material Engineering, Quzhou University, Quzhou 324000, China

Contents

Fig. S1. (a) Thermogravimetric (TG) and (b) derivative thermogravimetric (DTG) curves of the pristine PV matrix and the series of xBi-ZnO@PV (x = 0, 1, 3, 5, and 7) composites..2

Fig. S2. (a, b) SEM images of the 1Bi-ZnO@PV composite; (c) Corresponding EDS elemental mapping images (C, O, Si, Zn, and Bi). 2

Fig. S3. (a, b) SEM images of the 3Bi-ZnO@PV composite; (c) corresponding EDS elemental mapping images (C, O, Si, Zn, and Bi). 2

Fig. S4. (a, b) SEM images of the 7Bi-ZnO@PV samples; (c) corresponding EDS elemental mapping images (C, O, Si, Zn, Bi)..3

Fig. S5. (a) N_2 adsorption-desorption isotherms and (b) the corresponding BJH pore size distribution curves of the pristine PV, 0Bi-ZnO(1.0)@PV, and 5Bi-ZnO@PV samples. 3

Fig. S6. (a) VB-XPS spectrum and (b) UPS secondary-electron cutoff region of 5Bi-ZnO@PV. 3

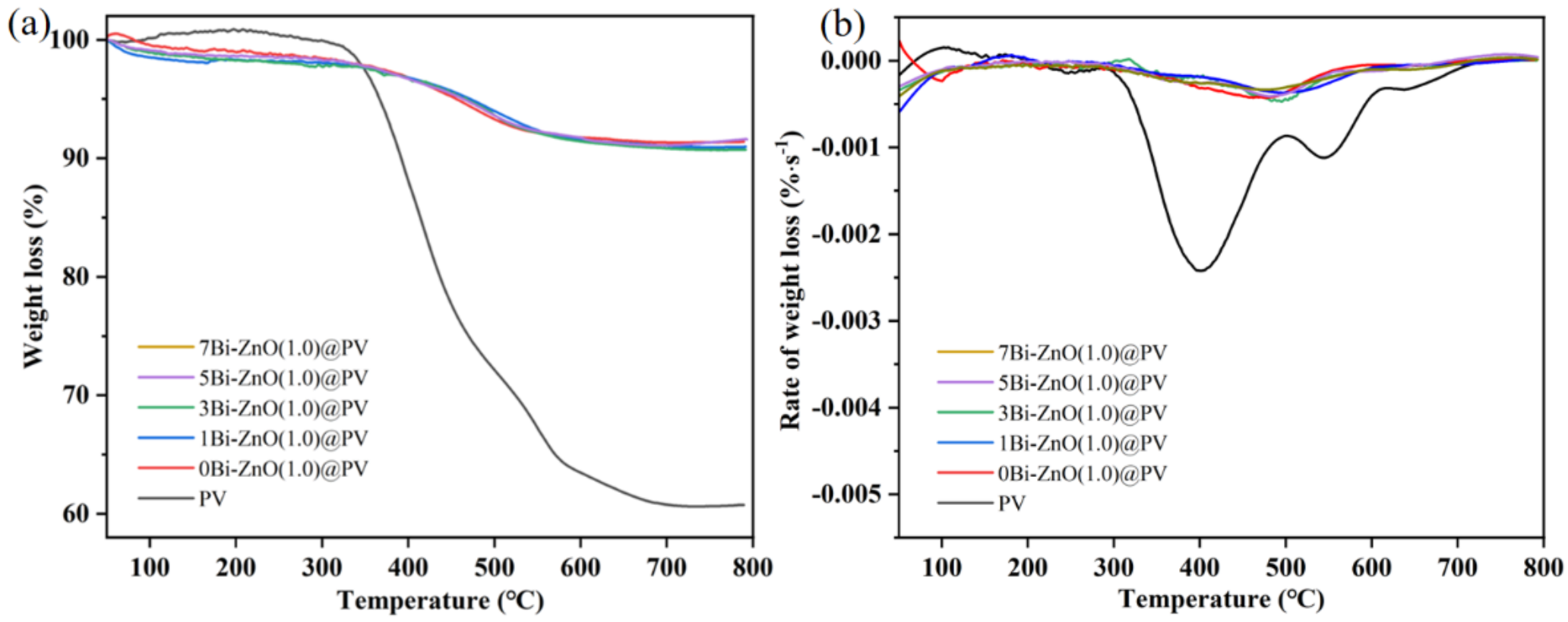

Fig. S1. (a) Thermogravimetric (TG) and (b) derivative thermogravimetric (DTG) curves of the pristine PV matrix and the series of xBi-ZnO@PV (x = 0, 1, 3, 5, and 7) composites.

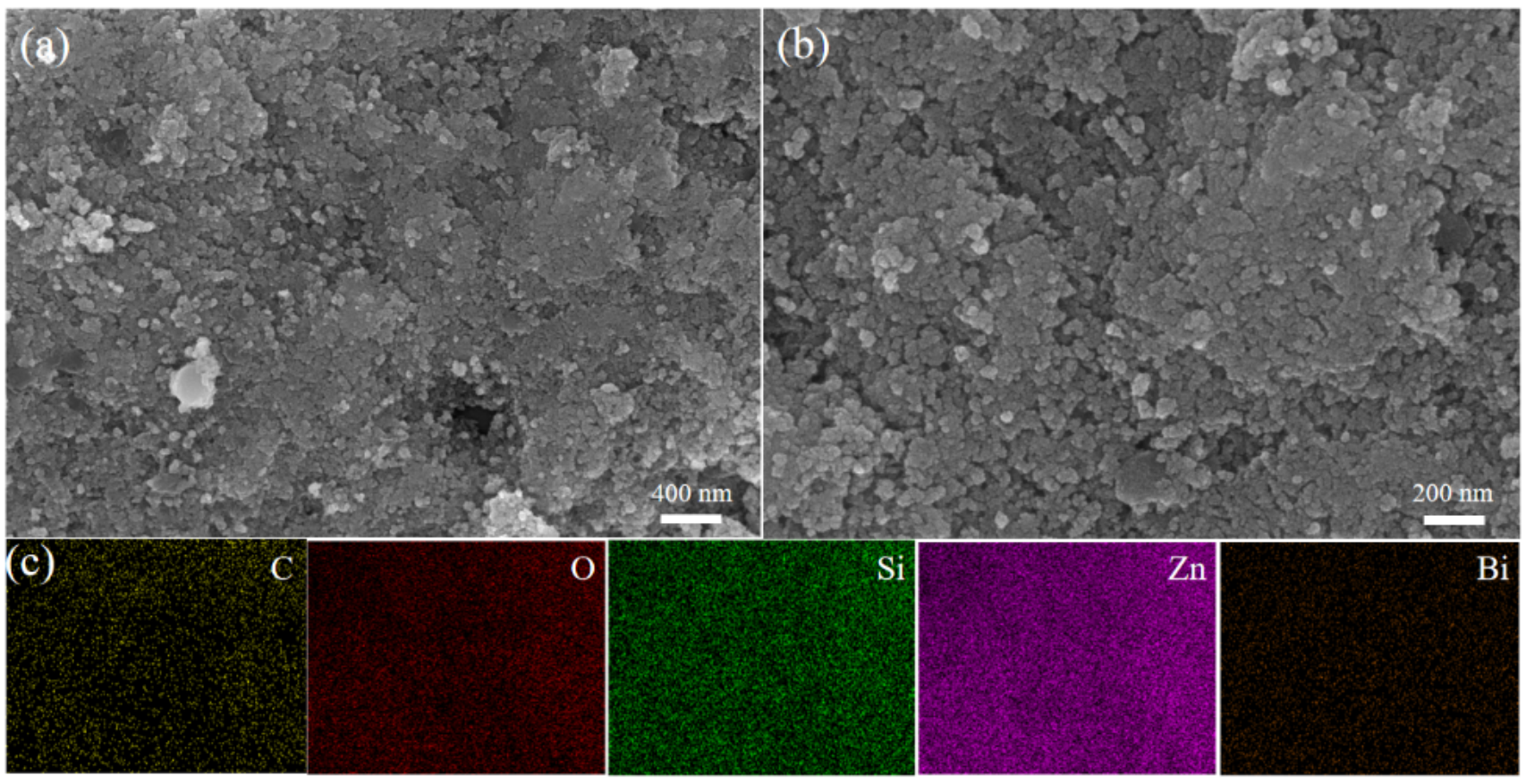

Fig. S2. (a, b) SEM images of the 1Bi-ZnO@PV composite; (c) Corresponding EDS elemental mapping images (C, O, Si, Zn, and Bi).

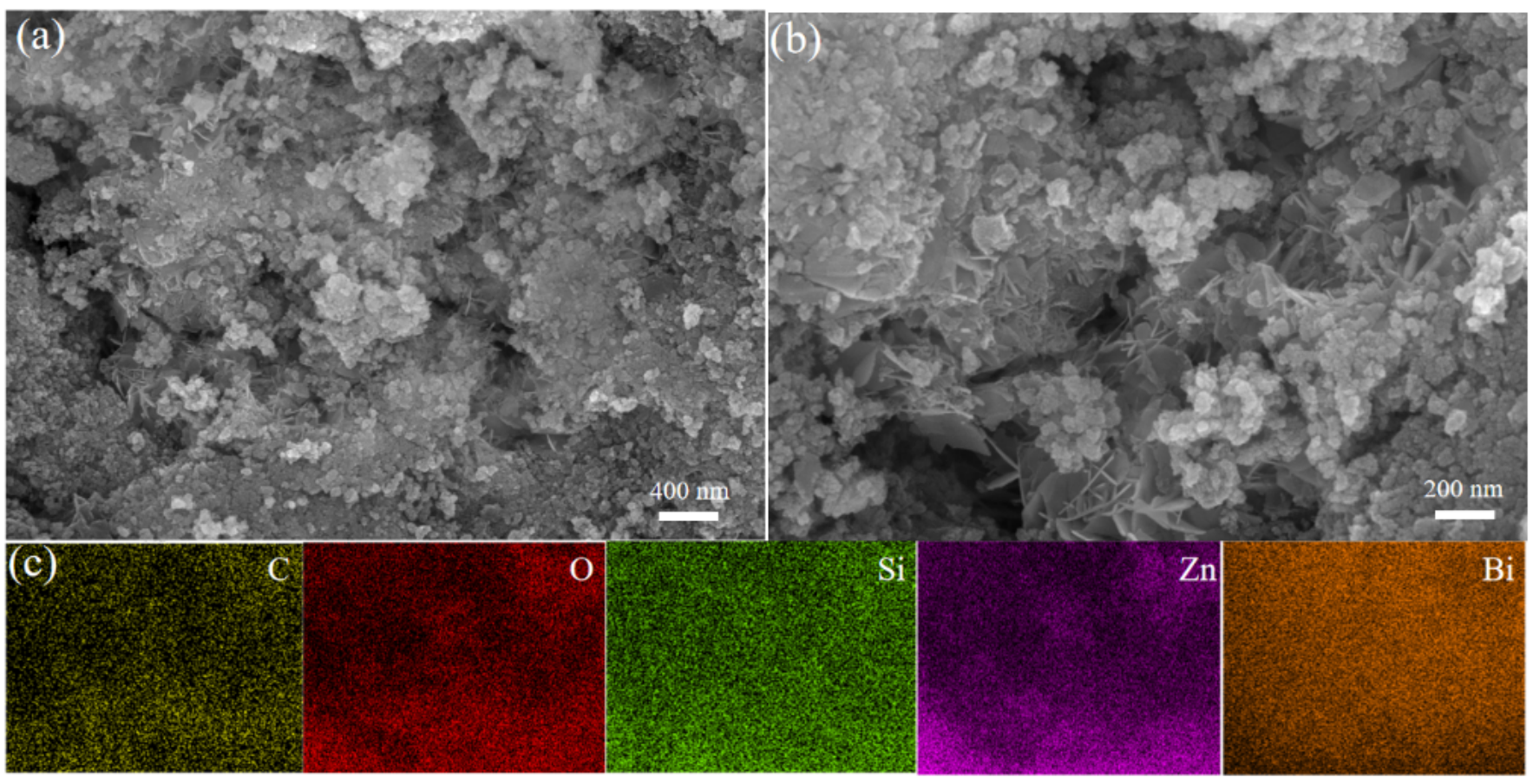

Fig. S3. (a, b) SEM images of the 3Bi-ZnO@PV composite; (c) corresponding EDS elemental mapping images (C, O, Si, Zn, and Bi).

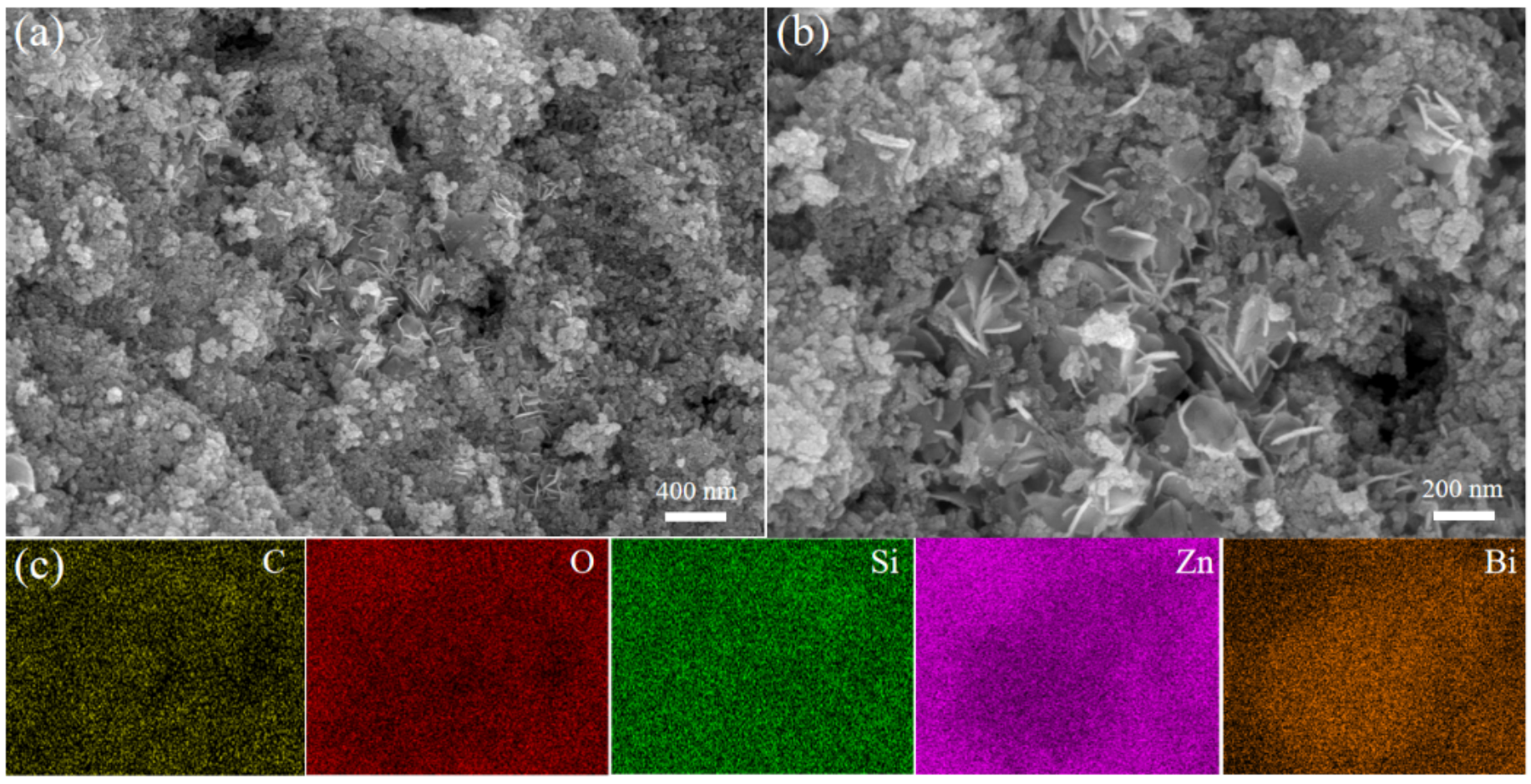

Fig. S4. (a, b) SEM images of the 7Bi-ZnO@PV samples; (c) corresponding EDS elemental mapping images (C, O, Si, Zn, Bi).

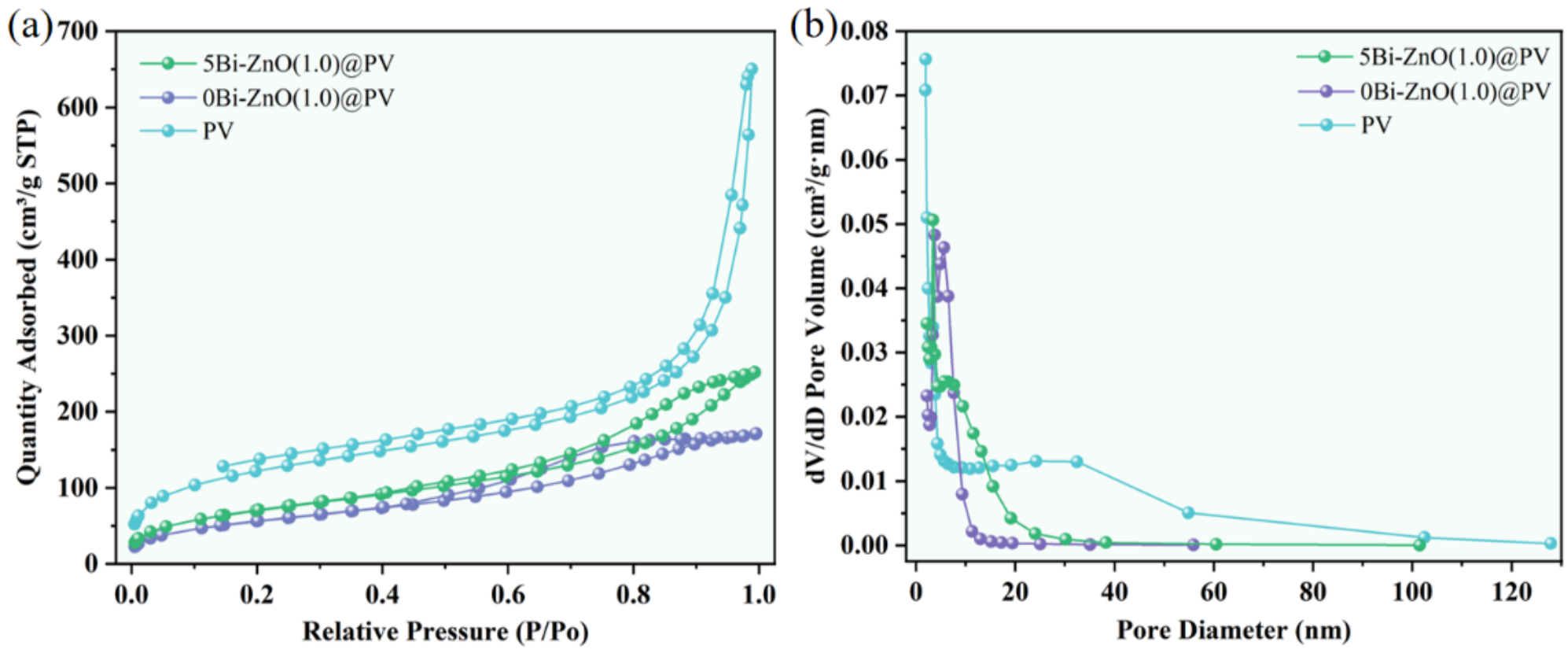

Fig. S5. (a) N_2 adsorption-desorption isotherms and (b) the corresponding BJH pore size distribution curves of the pristine PV, 0Bi-ZnO(1.0)@PV, and 5Bi-ZnO@PV samples.

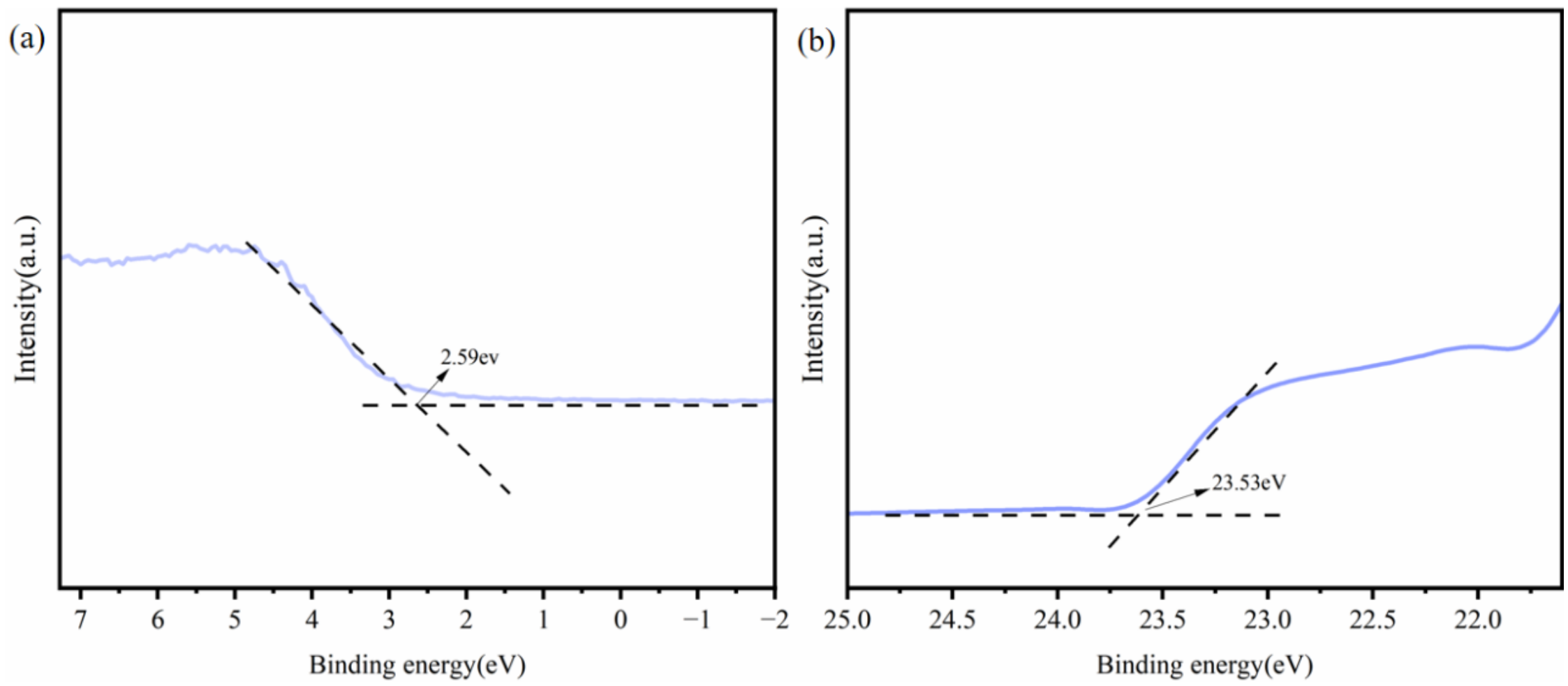

Fig. S6. (a) VB-XPS spectrum and (b) UPS secondary-electron cutoff region of

5Bi-ZnO@PV.
